# Estimates of Type 2 Diabetes Mellitus Burden Attributable to Particulate Matter Pollution and Its 30-Year Change Patterns: A Systematic Analysis of Data From the Global Burden of Disease Study 2019

**DOI:** 10.3389/fendo.2021.689079

**Published:** 2021-08-13

**Authors:** Ying Wu, Rongguo Fu, Chen Lei, Yujiao Deng, Weiyang Lou, Li Wang, Yi Zheng, Xinyue Deng, Si Yang, Meng Wang, Zhen Zhai, Yuyao Zhu, Dong Xiang, Jingjing Hu, Zhijun Dai, Jie Gao

**Affiliations:** ^1^Department of Nephrology, The Second Affiliated Hospital of Xi’an Jiaotong University, Xi’an, China; ^2^Department of Endocrinology, The General Hospital of Ningxia Medical University, Yinchuan, China; ^3^Department of Oncology, The Second Affiliated Hospital of Xi’an Jiaotong University, Xi’an, China; ^4^Department of Breast Surgery, The First Affiliated Hospital, College of Medicine, Zhejiang University, Hangzhou, China; ^5^Celilo Cancer Center, Oregon Health Science Center affiliated Mid-Columbia Medical Center, The Dalles, OR, United States; ^6^Dana-Farber Cancer Institute, Harvard Medical School, Boston, MA, United States

**Keywords:** type 2 diabetes mellitus, PM_2.5_ pollution, ambient particulate matter pollution, household air pollution, Global Burden of Disease

## Abstract

**Background:**

Epidemiological trends of type 2 diabetes mellitus attributable to fine particulate matter (PM_2.5_) pollution remain unclear. Here, we estimated spatiotemporal trends of type 2 diabetes mellitus burden attributable to PM_2.5_ pollution, including ambient particulate matter pollution (APMP) and household air pollution (HAP), from 1990–2019.

**Methods:**

Data were obtained from the Global Burden of Disease Study 2019 and were analyzed by age, sex, year, and location. Joinpoint regression analysis was applied in the analysis of temporal trends in type 2 diabetes mellitus burden over the 30 years.

**Results:**

Globally, PM_2.5_ pollution contributed to 292.5 thousand deaths and 13 million disability-adjusted life-years (DALYs) in 2019. APMP ranked third among all risk factors, causing an increase in type 2 diabetes mellitus burden from 1990, whereas the impact of HAP significantly fell during the same period. Both APMP and HAP contributed the most to deaths and DALYs of type 2 diabetes mellitus among older people. However, the age-standardized death and DALY rates of type 2 diabetes mellitus attributable to APMP were greater among males and people in the middle socio-demographic index countries, especially in Southern Sub-Saharan Africa. For HAP, type 2 diabetes mellitus burden was modestly higher in females and was highest in Oceania, which was the only region with an increase from 1990.

**Conclusions:**

PM_2.5_ pollution resulted in substantial and increasing type 2 diabetes mellitus burden worldwide. Hence, governments and health systems should take steps to reduce air pollution to mitigate this increasing burden.

## Introduction

Diabetes was the fifth leading cause of global deaths in 2019, with a prevalence of 463 million that is projected to reach 548 million in 2045 (1, 2). Type 2 diabetes mellitus is the most common type of diabetes, accounting for >90% of the total number of cases, and is the fifth leading cause of death for people aged 50–74 years (1). This leads to a great number of consequent complications, lowering the quality of life and functional capacities of patients (3).

According to estimates, the burden of diabetes and its consequent economic costs will continuously increase up to 2030, even if the targets of the Sustainable Development Goals and World Health Organization Global Action Plan were met (4). Thus, it is imperative to take measures to reduce its modifiable risk factors, such as unhealthy lifestyles and obesity, which are associated with an increased risk of type 2 diabetes mellitus (5). Accumulating evidence suggests that air pollution is also a risk factor for type 2 diabetes mellitus and could affect its clinical outcomes, including increasing the risk of death (6, 7). Air pollution has also been listed as one of the risk factors for various diseases, consisting of gases and different particles in the air, of which fine particulate matter (PM_2.5_) pollution accounted for >90% of public health impact (8). The link between ambient PM_2.5_ pollution and type 2 diabetes mellitus has been verified in previous studies. In one meta-analysis, six out of ten studies suggested that a 6%–49% increase in mortality was related to each 10 μg/m^3^ increment in ambient PM_2.5_ concentration (9). Additionally, ambient PM_2.5_ pollution exposure is also linked to higher fasting blood glucose, HbA1c, and the homeostatic model assessment of insulin resistance (9). A previous study also showed that PM_2.5_ can cause systematic inflammation, increase oxidative stress, and impair endothelial function (10). Furthermore, animal studies also demonstrated that PM_2.5_ exposure is associated with insulin resistance, hence inducing diabetes (11).

The Global Burden of Disease (GBD) Study 2017 has listed both outdoor and indoor PM_2.5_ pollution as risk factors for type 2 diabetes mellitus (12). However, no study has investigated the epidemiological pattern of type 2 diabetes mellitus burden attributable to PM_2.5_ pollution. Thus, based on the latest data and improved methodologies of GBD 2019, we estimated the spatiotemporal trends of PM_2.5_-related type 2 diabetes mellitus burden and identified the highly affected regions, providing insight to assist policymaking and recommending actions to reduce air pollution and its corresponding disease burden.

## Materials and Methods

### Data Source

Data on the global burden of type 2 diabetes mellitus attributable to particulate matter pollution were obtained from GBD 2019. GBD studies provide worldwide and comprehensive assessments of health loss for 329 diseases across 204 countries and territories that are classified into 21 regions according to epidemiological similarities and geographical proximity, and into five groups based on the socio-demographic index (SDI; low, low-middle, middle, high-middle, and high SDI). The SDIs of countries are estimated based on economic growth, fertility rate, and educational attainment. The number of deaths, disability-adjusted life-years (DALYs), and corresponding age-standardized rates (ASRs) attributable to particulate matter pollution from 1990 to 2019 were extracted and analyzed by age, sex, location, and year.

### Estimation of Particulate Matter Pollution Exposure and Its Attributable Type 2 Diabetes Mellitus Burden

In GBD 2019, particulate matter pollution includes outdoor and indoor PM_2.5_ pollution, and PM_2.5_ refers to particles with an aerodynamic diameter ≤2.5 μm. GBD 2019 identified different PM_2.5_ sources: outdoor PM_2.5_ pollution, also called ambient particulate matter pollution (APMP), owing to exposure to PM_2.5_ in the outdoor air, and indoor PM_2.5_ pollution, also called household air pollution (HAP) from solid fuels, which refers to exposure to PM_2.5_ owing to the use of solid cooking fuels (wood, coal, charcoal, agricultural residues, and dung). A uniform distribution of the theoretical minimum risk exposure level between 2.4 µg/m^3^ and 5.9 µg/m^3^ for both APMP and HAP represents the level that minimizes risk at the population level or the level that captures the maximum attributable burden. In GBD studies, the attribution of type 2 diabetes mellitus deaths and DALYs to particulate matter pollution was calculated by applying the population-attributable fraction for age, sex, location, and year. Further details regarding estimation of particulate matter pollution exposure and its attributable type 2 diabetes mellitus burden have been provided in the previous study (1).

### Statistical Analyses

We analyzed all the attributable burdens by age, sex, year, and location to determine the impact of particulate matter pollution on type 2 diabetes mellitus. When comparing different populations or the same population in different periods, we used the ASRs of deaths and DALYs to eliminate the effects caused by differences in population structures. All cases and their corresponding ASRs per 100,000 people were reported with 95% uncertainty intervals (UIs). The R program (R Core Team, version 3.5.2, Vienna, Austria) was used to perform all the above-mentioned analyses.

The Joinpoint regression model, a set of statistically linear models, was used to evaluate the temporal trends in age-standardized death and DALY rates. Changes in trends were described by connecting several line segments on a logarithmic scale at the “joinpoints” and identifying points where the trend linear slope significantly changed over time (13). Joinpoint regression analysis was conducted using the Joinpoint software (version 4.7.0) from the Surveillance Research Program of the US National Cancer Institute. Annual percentage changes (APCs) and their 95% confidence intervals were also calculated. The p-value was estimated with a significance level of 0.05.

### Data and Resource Availability

All data included in this study can be accessed through the Global Health Data Exchange query tool (http://ghdx.healthdata.org/gbd-results-tool).

## Results

### Global Burden of Type 2 Diabetes Mellitus Attributable to Particulate Matter Pollution in 2019

Globally, a total of 66.3 million (55.5 to 79.0) DALYs and 1.5 million (1.4 to 1.6) deaths of type 2 diabetes mellitus were documented due to 17 risk factors in 2019 (**Table 1** and **Supplementary Figure 1**). Following high fasting plasma glucose, high body mass index contributed to 42.6% of total deaths and 51.9% of total DALYs of type 2 diabetes mellitus. However, particulate matter pollution accounted for 19.9% of total deaths and 19.6% of total DALYs of type 2 diabetes mellitus, among which APMP contributed to 13.4% of total deaths and 13.6% of total DALYs and HAP resulted in 6.5% of total deaths and 5.9% of total DALYs (**Supplementary Figure 1**). Thus, these two risk factors are posing a great challenge to public health.

**Table 1:** Global and regional type 2 diabetes mellitus burdens attributable to particulate matter pollution in 1990 and 2019.

	APMP				HAP			
	Deaths, in thousand (95% UI)		DALYs, in thousand (95% UI)		Deaths, in thousand (95% UI)		DALYs, in thousand (95% UI)	
	1990	2019	1990	2019	1990	2019	1990	2019
**Global**	55.82 (37.34 to 78.67)	196.79 (136.3 to 258.39)	2330.45 (1463.32 to 3317.95)	9034 (6135.35 to 12213.37)	74.8 (48.66 to 120.77)	95.74 (60.53 to 138.46)	3126.22 (1983.72 to 5000.79)	3921.79 (2431.56 to 5851.35)
Male	25.31 (16.47 to 35.94)	99.35 (69.73 to 131.6)	1172.26 (735.43 to 1676.67)	4820.52 (3300.59 to 6542.77)	34.44 (22.29 to 55.96)	42.89 (26.14 to 64.22)	1492.47 (936.51 to 2374.42)	1815.56 (1048.69 to 2775.46)
Female	30.52 (20.17 to 42.99)	97.44 (67.66 to 127.6)	1158.19 (727.1 to 1663.83)	4213.48 (2842.53 to 5763.9)	40.37 (26.3 to 65.74)	52.85 (34.14 to 78.29)	1633.75 (1028.93 to 2695.88)	2106.22 (1324.09 to 3162.26)
**SDI rank**								
High SDI	16.59 (9.84 to 24.39)	18.45 (11.43 to 27.43)	656.77 (372.56 to 991.78)	992.58 (575.98 to 1549.41)	0.74 (0.38 to 1.23)	0.15 (0.04 to 0.4)	30.93 (15.8 to 52.71)	6.54 (1.64 to 17.74)
High-middle SDI	16.82 (11.45 to 23.08)	41.09 (29.5 to 53.5)	722.24 (469.16 to 1027.09)	2021.31 (1381.3 to 2783.14)	8.08 (5.09 to 11.9)	4.31 (1.91 to 7.9)	399.11 (242.42 to 603.84)	207.79 (85.42 to 400.62)
Middle SDI	16.21 (10.12 to 23.9)	86.42 (61.62 to 111.19)	695.82 (413.83 to 1040.69)	3848.94 (2652.58 to 5172.93)	24.92 (16.69 to 35.26)	25.87 (14.23 to 41.33)	1093.17 (713.2 to 1583.44)	1045.8 (556.5 to 1708.18)
Low-middle SDI	4.64 (1.99 to 8.65)	41.24 (26.58 to 57.84)	194.27 (82.87 to 362.08)	1753.19 (1107.32 to 2486.21)	24.65 (15.81 to 41.8)	38.83 (24.72 to 56.58)	1008.03 (623.79 to 1771.22)	1602.92 (996.63 to 2372.75)
Low SDI	1.52 (0.44 to 3.31)	9.46 (4.97 to 15.2)	59.56 (17.54 to 131.94)	412.2 (220.01 to 660)	16.34 (9.73 to 32.5)	26.46 (17.38 to 41.74)	591.9 (352.69 to 1219.5)	1053.52 (661.56 to 1651.58)
**21 GBD regions**								
Andean Latin America	0.34 (0.15 to 0.61)	1.99 (1.28 to 2.78)	12.62 (5.45 to 21.85)	75.52 (48.02 to 104.5)	0.49 (0.3 to 0.72)	0.58 (0.29 to 0.95)	17.84 (10.79 to 26.05)	21.04 (10.25 to 35.26)
Australasia	0.14 (0.01 to 0.33)	0.21 (0.05 to 0.43)	4.58 (0.42 to 11.14)	8.67 (1.97 to 18.29)	0.01 (0 to 0.02)	0 (0 to 0.01)	0.27 (0.03 to 0.8)	0.07 (0.01 to 0.21)
Caribbean	0.79 (0.31 to 1.31)	1.84 (1.01 to 2.87)	29.94 (11.67 to 52.06)	78.18 (42.3 to 121.64)	1.01 (0.68 to 1.53)	1.04 (0.65 to 1.58)	34.73 (23.15 to 53.25)	43.01 (27.05 to 64.48)
Central Asia	0.43 (0.2 to 0.7)	2.84 (1.91 to 3.94)	23.07 (10.17 to 39.89)	135.91 (88.87 to 191.79)	0.42 (0.25 to 0.61)	0.48 (0.23 to 0.89)	22.45 (12.36 to 33.79)	23.64 (11.06 to 43.91)
Central Europe	2.55 (1.42 to 3.65)	4.27 (2.88 to 5.68)	128.18 (67.59 to 193.6)	233.55 (149.18 to 327.88)	1.07 (0.56 to 1.78)	0.62 (0.22 to 1.3)	52.87 (27.02 to 87)	30.87 (10.76 to 66.77)
Central Latin America	4.31 (2.3 to 6.52)	14.58 (9.94 to 19.66)	167.88 (89.05 to 258.99)	592.99 (390.05 to 818)	2.85 (1.76 to 4.19)	4.14 (2.38 to 6.53)	120.89 (75.01 to 176.62)	168.53 (98.49 to 267.34)
Central Sub-Saharan Africa	0.22 (0.06 to 0.52)	1.12 (0.56 to 1.88)	7.97 (2.22 to 18.54)	46.53 (23.58 to 79.33)	2 (1.22 to 3.62)	3.02 (1.88 to 4.57)	73.29 (44.41 to 133.95)	130.44 (82.59 to 200.34)
East Asia	5.49 (2.52 to 9.67)	34.12 (24.08 to 45.27)	315.4 (139.27 to 568.82)	1901.69 (1262.78 to 2611.41)	12.01 (7.6 to 17.68)	8.51 (4.4 to 14.46)	707.75 (432.08 to 1064.29)	465.42 (229.18 to 812.93)
Eastern Europe	1.59 (0.77 to 2.51)	2.23 (1.16 to 3.43)	109.89 (50.43 to 182.83)	133.41 (67.24 to 211.98)	0.26 (0.11 to 0.48)	0.09 (0.03 to 0.24)	17.06 (7.35 to 32.13)	5.86 (1.72 to 14.73)
Eastern Sub-Saharan Africa	0.39 (0.11 to 0.93)	1.93 (0.95 to 3.37)	12.57 (3.63 to 29.8)	67.72 (33.65 to 119.14)	7.52 (4.54 to 15.25)	10.71 (7.01 to 16.56)	244.64 (144.6 to 507.3)	374.22 (245.13 to 581.85)
High-income Asia Pacific	2.05 (0.85 to 3.27)	3.67 (2.38 to 4.89)	104.85 (39.88 to 171.97)	221.1 (134.85 to 325.09)	0.06 (0.02 to 0.13)	0 (0 to 0.01)	2.64 (0.85 to 6.42)	0.35 (0.07 to 1.07)
High-income North America	5.49 (2 to 9.61)	4.62 (2.09 to 8.23)	230.63 (80.38 to 409.75)	253.13 (109.32 to 454.46)	0.03 (0.01 to 0.07)	0.01 (0 to 0.05)	1.05 (0.28 to 2.76)	0.78 (0.13 to 2.54)
North Africa and Middle East	5.54 (3.85 to 7.4)	19.94 (14.36 to 25.7)	213.9 (146.41 to 285.15)	996.41 (710.33 to 1340.57)	2.67 (1.57 to 4.31)	1.42 (0.82 to 2.32)	107.56 (63.61 to 175.32)	76.57 (45.17 to 119.77)
Oceania	0.05 (0.01 to 0.13)	0.29 (0.08 to 0.66)	1.67 (0.45 to 4.61)	10.31 (3.03 to 23.78)	0.5 (0.33 to 0.8)	1.32 (0.84 to 1.94)	18.27 (11.92 to 29.5)	51.61 (33.28 to 75.12)
South Asia	5.36 (2.11 to 10.05)	51.45 (34.36 to 69.97)	245.22 (103.79 to 462.96)	2303.3 (1523.07 to 3186.33)	20.06 (12.34 to 35.2)	31.32 (19.3 to 47.82)	881.9 (527.56 to 1575.57)	1370.13 (811.06 to 2136.39)
Southeast Asia	4.46 (2.1 to 7.73)	23.63 (15.3 to 33.24)	165.22 (76.74 to 291.64)	899.57 (574.77 to 1290.99)	13.77 (9.11 to 19.95)	19.55 (11.83 to 28.79)	484.86 (318.51 to 711.98)	713.05 (424.91 to 1058.46)
Southern Latin America	0.91 (0.33 to 1.61)	1.92 (1.22 to 2.72)	29.83 (11.37 to 52.81)	78.25 (48.4 to 114.8)	0.36 (0.19 to 0.61)	0.11 (0.04 to 0.25)	12.1 (6.01 to 20.77)	4.5 (1.52 to 10.29)
Southern Sub-Saharan Africa	1.1 (0.73 to 1.53)	5.09 (3.52 to 6.7)	34.91 (22.56 to 48.39)	158.12 (107.2 to 210.6)	1.27 (0.86 to 1.81)	1.92 (1.15 to 2.89)	41.86 (27.81 to 58.77)	60.69 (36.13 to 90.99)
Tropical Latin America	1.76 (0.73 to 3.12)	5.68 (3.44 to 8.64)	71.9 (30.23 to 126.73)	221.76 (128.22 to 339.74)	2.67 (1.7 to 3.82)	2.08 (1 to 3.65)	100.92 (64.01 to 143.93)	73.14 (33.26 to 132.33)
Western Europe	12.02 (5.92 to 18.47)	10.05 (6.13 to 14.82)	393.49 (185.96 to 625.14)	441.35 (250.96 to 688.76)	0.31 (0.12 to 0.66)	0.05 (0.01 to 0.14)	9.98 (3.78 to 22.09)	2.15 (0.56 to 5.82)
Western Sub-Saharan Africa	0.86 (0.31 to 1.73)	5.33 (3.04 to 8.26)	26.75 (9.58 to 54.91)	176.54 (99.53 to 277.04)	5.47 (3.14 to 10.87)	8.76 (5.39 to 13.81)	173.3 (101.11 to 341.9)	305.71 (184.88 to 488.72)

DALY, disability-adjusted life-year; APMP, ambient particulate matter pollution; HAP, household air pollution from solid fuels; GBD, global burden of disease; SDI, socio-demographic index; UI, uncertainty interval.

More specifically, in 2019, APMP contributed to 196.8 thousand (136.3 to 258.4) deaths and 9.0 million (6.1 to 12.2) DALYs of type 2 diabetes mellitus worldwide (**Table 1**). By contrast, the HAP-attributable deaths and DALYs of type 2 diabetes mellitus were relatively lower, at 95.7 thousand (60.5 to 138.5) deaths and 3.9 million (2.4 to 5.9) DALYs in 2019. The age-standardized death and DALY rates of type 2 diabetes mellitus attributable to APMP were 2.5/100,000 (1.7 to 3.2) and 109.0/100,000 (74.1 to 147.2), respectively—more than doubled the age-standardized rates of type 2 diabetes mellitus due to HAP in 2019 (**Supplementary Table 1**).

### Temporal Trends of Type 2 Diabetes Mellitus Burdens Attributable to Particulate Matter Pollution

**Figure 1:**
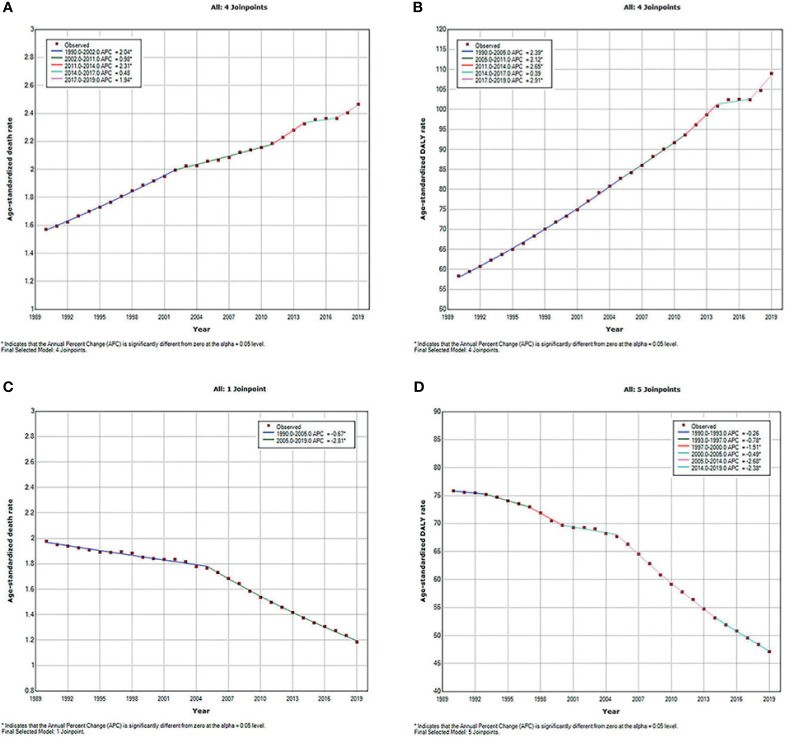
Temporal trends of global type 2 diabetes mellitus burden attributable to particulate matter pollution from 1990 to 2019 for both sexes combined for all ages. **(A)** ASDR of type 2 diabetes mellitus attributable to APMP; **(B)** Age-standardized DALY rate of type 2 diabetes mellitus attributable to APMP; **(C)** ASDR of type 2 diabetes mellitus attributable to HAP; **(D)** Age-standardized DALY rate of type 2 diabetes mellitus attributable to HAP. ASDR, age-standardized death rate; APMP, ambient particulate matter pollution; DALY, disability-adjusted life-year; HAP, household air pollution.

The type 2 diabetes mellitus deaths and DALYs due to APMP and HAP exposure increased significantly since 1990, but the age-standardized death and DALY rates due to these two risk factors presented different trends. As shown in **Figure 1**, the APMP-attributable ASDR of type 2 diabetes mellitus increased with different APCs, and the most significant increase took place between 2011 and 2014 (APC = 2.31%, *p* < 0.05). The age-standardized DALY rate also rose rapidly with different APCs from 1990, reaching an approximately doubled burden in 2019, and the most remarkable increase was between 2017 and 2019 (APC =2.91%, *p* < 0.05). For HAP, the ASDR of type 2 diabetes mellitus decreased steadily from 1990 to 2005 and then experienced a significant decline until 2019, with an APC of 2.81% (*p* < 0.05). Similarly, the age-standardized DALY rate of type 2 diabetes mellitus markedly fell from 2005 to 2019 (2005–2014: APC = 2.68%, *p* < 0.05; 2014–2019: APC = 2.38%, *p* < 0.05).

### Age- and Sex-Specific Type 2 Diabetes Mellitus Burden Attributable to Particulate Matter Pollution

**Figure 2:**
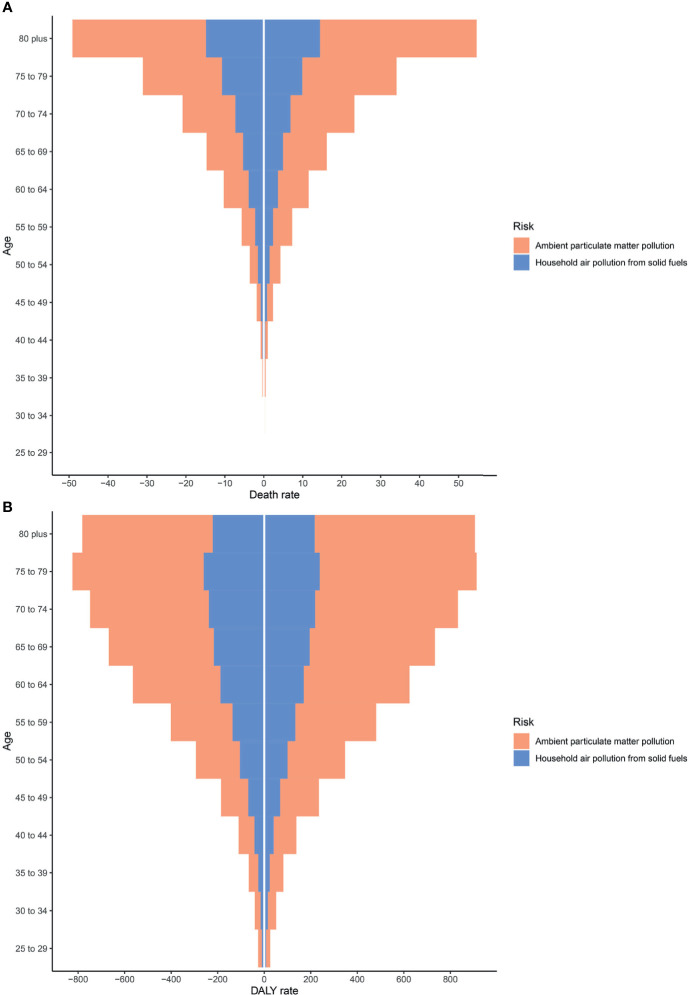
Type 2 diabetes mellitus burden among different genders and ages in 2019. **(A)** Death rates. **(B)** DALY rates. DALY, disability-adjusted life year.

As shown in **Supplementary Figure 2**, APMP and HAP exposure have the greatest contributions to type 2 diabetes mellitus burdens among people aged 50–74 years. From 1990 to 2019, the APMP-attributable burden of type 2 diabetes mellitus in all ages presented substantial increases since 1990, whereas the HAP-attributable burden increased only among older people and remained stable among people aged <49 years. In the subgroup analysis of genders, the total burden of type 2 diabetes mellitus due to particulate matter pollution among males was higher than that in females, whereas the distributions of burdens attributable to APMP and HAP in different ages were similar between females and males (**Figure 2**). For both genders, the death rates of type 2 diabetes mellitus attributable to APMP and HAP increased with age, peaking among people aged >80 years, whereas DALY rates were highest among people aged 75–79 years (**Figure 2**).

### Regional and National Type 2 Diabetes Mellitus Burden Attributable to Particulate Matter Pollution

**Figure 3:**
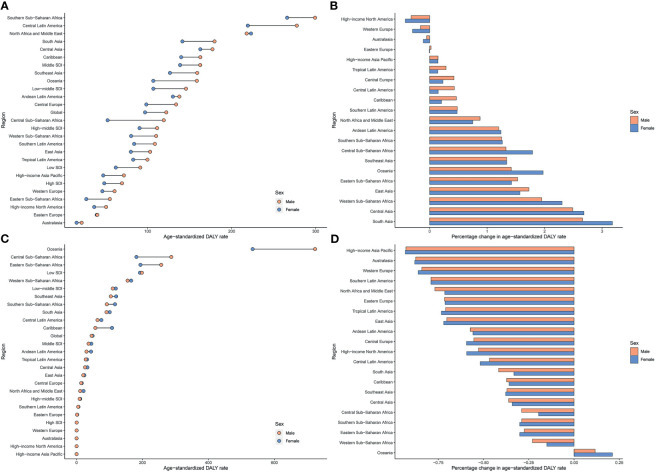
Age-standardized DALY rates of type 2 diabetes mellitus attributable to particulate matter pollution in 2019 and their percentage changes in rates from 1990 to 2019 across 21 GBD regions among different genders. **(A)** Age-standardized DALY rates of type 2 diabetes mellitus attributable to APMP; **(B)** Percentage changes in age-standardized DALY rates attributable to APMP from 1990 to 2019; **(C)** Age-standardized DALY rates of type 2 diabetes mellitus attributable to HAP; **(D)** Percentage changes in age-standardized DALY rates attributable to HAP from 1990 to 2019. APMP, ambient particulate matter pollution; DALY, disability-adjusted life-year; HAP, household air pollution.

**Figure 4:**
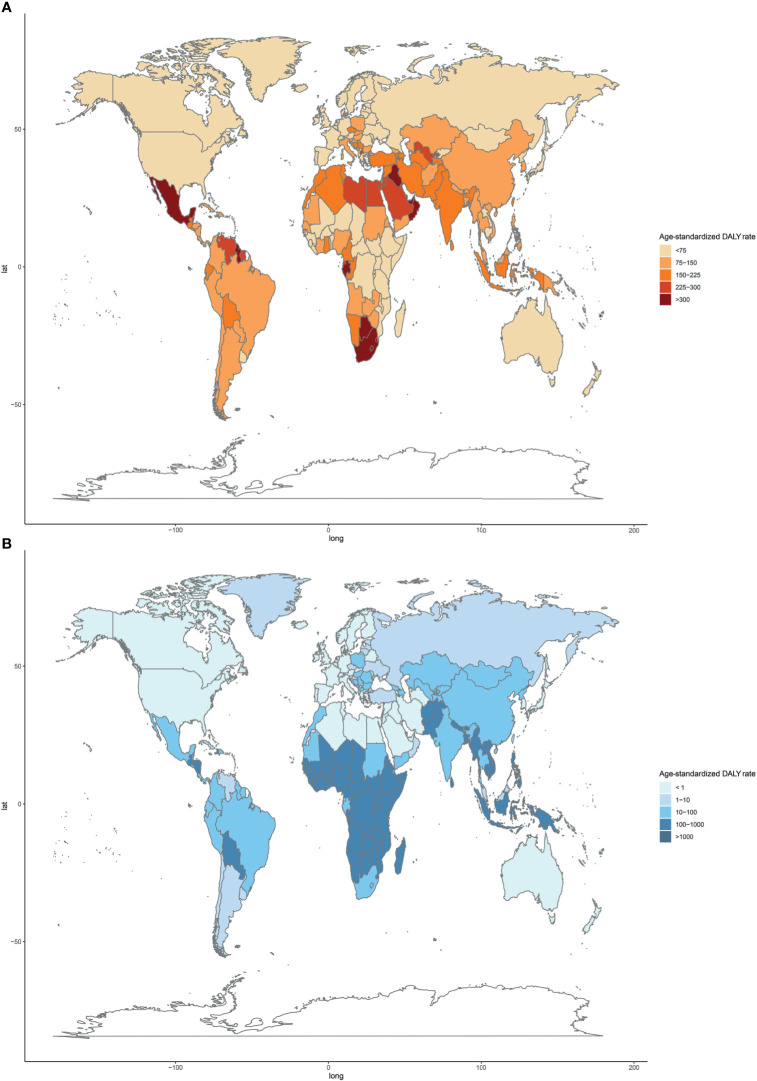
Age-standardized DALY rates of type 2 diabetes mellitus attributable to particulate matter pollution among 204 countries and territories in 2019. **(A)** APMP; **(B)** HAP. DALY, disease-adjusted life-year; APMP, ambient particulate matter pollution; HAP, household air pollution.

Regionally, Southern Sub-Saharan Africa had the highest age-standardized DALY and death rates of type 2 diabetes mellitus due to APMP exposure in 2019 (**Figure 3**; **Supplementary Figure 3** and **Supplementary Table 1**). However, South Asia had the greatest increase in age-standardized DALY rate of type 2 diabetes mellitus due to APMP from 1990, whereas Central Asia had the largest increase in ASDR. By contrast, the APMP-attributable age-standardized death and DALY rates of type 2 diabetes mellitus decreased significantly in High-income North America, Western Europe, and Australasia during the last 30 years (**Figure 3** and **Supplementary Figure 3**). At the national level, in 1990, the age-standardized death and DALY rates of type 2 diabetes mellitus attributable to APMP were greatest in Qatar compared to other nations, whereas up to 2019, Bahrain showed the highest rates, followed by Qatar (**Figure 4** and **Supplementary Figure 4**). By contrast, in 2019, the lowest ASDR of type 2 diabetes mellitus due to APMP exposure was observed in Finland, and Iceland had the lowest age-standardized DALY rate. During the past 30 years, Cabo Verde had the greatest increase in ASDR of type 2 diabetes mellitus due to APMP exposure, and Equatorial Guinea showed the largest increase in age-standardized DALY rate (**Supplementary Table 2**). However, Singapore and Sweden showed the greatest decrease in age-standardized death and DALY rates, respectively (**Supplementary Table 2**).

For HAP, in 2019, the age-standardized DALY and death rates of type 2 diabetes mellitus were highest in Oceania, with more than double the burden of Eastern Sub-Saharan Africa where had the second-highest age-standardized burden due to HAP (**Figure 3**; **Supplementary Figure 3** and **Supplementary Table 1**). However, Oceania was the only region with an increase in age-standardized death and DALY rates of type 2 diabetes mellitus attributable to HAP since 1990. Nationally, Kiribati showed the highest HAP-attributable age-standardized DALY and death rates of type 2 diabetes mellitus in 1990 and 2019 (**Figure 4** and **Supplementary Figure 4**). However, the lowest ASDR was recorded in Monaco in 1990 and 2019, whereas United Arab Emirates showed the lowest age-standardized DALY rate attributable to HAP in 2019. During the past 30 years, the greatest increase in age-standardized death and DALY rates of type 2 diabetes mellitus were in Guatemala, whereas the largest declines were in Saudi Arabia (**Supplementary Table 2**).

### Association of Type 2 Diabetes Mellitus Burden Attributable to Particulate Matter Pollution and SDI

**Figure 5:**
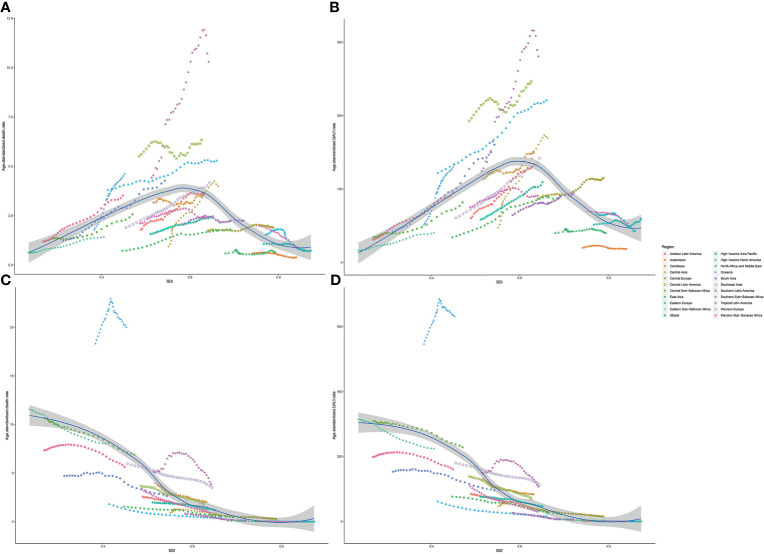
Association between type 2 diabetes mellitus burden attributable to particulate matter pollution and SDI among 21 regions. **(A)** ASDR of type 2 diabetes mellitus attributable to APMP; **(B)** Age-standardized DALY rate of type 2 diabetes mellitus attributable to APMP; **(C)** ASDR of type 2 diabetes mellitus attributable to HAP; **(D)** Age-standardized DALY rates of type 2 diabetes mellitus attributable to HAP. ASDR, age-standardized death rate; DALY, disability-adjusted life-year; SDI, socio-demographic index; APMP, ambient particulate matter pollution; HAP, household air pollution.

The age-standardized death and DALY rates of type 2 diabetes mellitus attributable to particulate matter pollution varied greatly with SDI. As shown in **Figure 5**, age-standardized death and DALY rates of type 2 diabetes mellitus due to APMP increased when SDI was <0.6, and then decreased with SDI, indicating the greatest burdens showing in countries with middle SDI (**Figure 5**). By contrast, the age-standardized death and DALY rates attributable to HAP steadily decreased with SDI, representing higher SDI countries showing lower burden (**Figure 5**). Additionally, during the past 30 years, APMP-attributable type 2 diabetes mellitus burden only decreased in high SDI countries, whereas the countries with low-to-middle SDI presented the biggest increase (**Table 1** and **Supplementary Table 1**). However, since 1990, the HAP-attributable burden decreased among all SDI quintiles, and the countries with low SDI showed the greatest increase (**Table 1** and **Supplementary Table 1**).

## Discussion

In this study, we estimated the global type 2 diabetes mellitus burden attributable to PM_2.5_ pollution, including APMP and HAP, along with its 30-year patterns. Over the last 30 years, the absolute number of deaths and DALYs attributable to both APMP and HAP significantly increased. This increase might be ascribed to two possible reasons: first, the increasing global population-weighted PM_2.5_, which rapidly increased from 2010 to 2015 and reached 44.2 µg/m^3^ in 2015 (14), and second, the growth and aging of the population, which stimulate the aggravation of air pollution and increasing disease burden (15, 16). However, the age-standardized death and DALY rates of type 2 diabetes mellitus due to exposure to these two risk factors presented different trends: for APMP, the age-standardized death and DALY rates markedly rose since 1990, whereas that due to HAP exposure significantly declined. There are some reasons for this adverse trend. First, the increase in APMP-attributable disease burden might be due to coal burning, urbanization, and the rising number of vehicles and industrial factories, which vary among different countries (17). Second, in recent years, owing to the change toward cleaner fuel and technological processes, HAP levels have been substantially declining, thus having decreasing disease burden (18). Nonetheless, APMP and HAP are still posing a huge health burden worldwide.

Generally, obesity and uneven diet were considered as part of risk factors for type 2 diabetes mellitus. However, sleep quality, hypertension and smoking were also found to be strongly associated with an elevated risk of type 2 diabetes mellitus (19). However, as our results showed, APMP and HAP ranked third and seventh respectively for type 2 diabetes mellitus deaths among all risk factors, the impact of which was more profound than some behavior risks, such as smoking and dietary risks. In prediabetes patients, PM_2.5_ was suggested to be associated with elevated fasting glucose, the release of pro-inflammation cytokines, higher blood pressure, and impairment of endothelial function (20). Meanwhile, a great amount of evidence has previously shown that PM_2.5_ can induce system inflammation and increase oxidative stress, leading to the release of cytokines and alteration in glucose homeostasis (10, 21, 22). Our results suggest that both APMP and HAP greatly contributed to the disease among elderly people. Similarly, according to the latest report of the International Diabetes Federation, the increasing prevalence with age is associated with a 19.9% (112 million) diabetes prevalence among people aged 65–79 years (2). PM_2.5_ exposure and aging have a complex interplay in diabetes among the elderly. Advanced age could exacerbate the chronic system inflammation and oxidative stress led by PM_2.5_, and aging is associated with reduced insulin secretion as well as insulin resistance due to the promotion of beta-cell death (23, 24).

For different sexes, our results showed that the type 2 diabetes mellitus burdens attributable to APMP were higher among males than females, whereas that attributable to HAP was slightly higher in females than in males. These differences might be associated with the roles of the different genders in society and within the family. In most cultures, males tend to have higher exposure to outdoor air pollution because of longer travel distances and work in industries that might use coal or other solid fuels, whereas females are traditionally responsible for more indoor housework and domestic cooking (25). However, a prior study suggested that elderly women tend to be more vulnerable to air pollution, particularly overweight women, which could increase the risk of diabetes (26). With the increasing numbers of the aging population as well as the increasing trend of women working outside, the burden of type 2 diabetes mellitus attributable to PM_2.5_ pollution for both genders should be paid better attention.

The type 2 diabetes mellitus burden attributable to APMP and HAP varied substantially across regions and nations. As our results illustrate, the middle SDI countries had the highest-burden attributable to APMP, whereas the low SDI countries contributed to the largest HAP-attributable burden. The Lancet Commission on air pollution previously suggested that over 90% of pollution-related deaths are recorded in low- and middle-income countries (27). More specifically, Southern Sub-Saharan Africa had the highest age-standardized death and DALY rates attributable to APMP, whereas South Asia had the highest absolute numbers of deaths and DALYs. As reported, the top three countries with the highest exposure to PM_2.5_ were in South Asia, whose population is exposed to severe air pollution due to garbage burning, fuel combustion, and industrial emissions (28). The changing profiles of population structure, urbanization, and westernized lifestyles in Southern Sub-Saharan Africa have also been associated with the increasing PM_2.5_ pollution and the corresponding growth in type 2 diabetes mellitus burden (29, 30). For HAP, the highest and only increase in burden from 1990 was recorded in Oceania (not including Australia and New Zealand), and 6 out of the top 10 countries with the highest type 2 diabetes mellitus burden attributable to HAP were in Oceania. Measures such as the transition to cleaner energy and improving the emission control system can help reduce the high level of indoor and outdoor air pollution (31). Policies aimed at reducing air pollution should be urgently devised and implemented, particularly in these regions.

To the best of our knowledge, this study is the first comprehensive analysis of spatiotemporal trends of type 2 diabetes mellitus burden attributable to PM_2.5_ pollution. Previous studies on this topic have explored the relationship between type 2 diabetes mellitus and air pollution but did not reveal the heavy disease burden due to air pollution and its distribution on a global scale. Our study provides a novel insight for countries and local health organizations to take measures to mitigate type 2 diabetes mellitus burden and its corresponding economic burden by reducing the modifiable risk factors of the disease. Nevertheless, although great efforts have been made to provide a comprehensive study, some limitations still exist in our analysis. First, the joint effects of PM_2.5_ and fasting blood glucose can lead to complex interactions (32), and HAP could also contribute to APMP, which should not be neglected. These interplays of various risk factors can cause overestimation or underestimation of the burden attributable to a single risk factor. Second, the data from some lower SDI countries and regions were insufficient, limiting the interpretation ability of our study and possibly causing underestimation of the effects of particulate matter pollution on disease burden.

To conclude, this study documents the increasing global burden of type 2 diabetes mellitus attributable to particulate matter pollution, from 1990 to 2019. Disease burdens due to particulate matter pollution exposure were higher among old people and people in low- and middle-SDI countries and regions, posing a huge burden on human health and a social cost on health care. Our study provides evidence regarding the heavy type 2 diabetes mellitus burden resulting from high levels of air pollution, identifies the highly affected areas, and can assist in establishing targeted programs and implementing effective measures to mitigate air pollution. Further studies focusing on strategies for controlling air pollution and avoiding heavy disease burden are urgently needed.

## Data Availability Statement

The original contributions presented in the study are included in the article/**Supplementary Material**. Further inquiries can be directed to the corresponding author.

## Author Contributions

JG and ZD conceived this study and took responsibility for manuscript review. YW analyzed the data and wrote the first draft, and RF and LC were responsible for reviewing the manuscript and data visualization. YD and WL analyzed the data. LW reviewed the manuscript. YZ, XD, and SY took responsibility for data analysis and visualization. MW, ZZ, and YYZ were responsible for methodologies. DX and JH reviewed the manuscript. All authors contributed to the article and approved the submitted version.

## Conflict of Interest

The authors declare that the research was conducted in the absence of any commercial or financial relationships that could be construed as a potential conflict of interest.

## Publisher’s Note

All claims expressed in this article are solely those of the authors and do not necessarily represent those of their affiliated organizations, or those of the publisher, the editors and the reviewers. Any product that may be evaluated in this article, or claim that may be made by its manufacturer, is not guaranteed or endorsed by the publisher.

## Supplementary Material

Supplementary Figure 1 Ranking changes of all risk factors for type 2 diabetes mellitus from 1990 to 2019 for both sexes combined for all ages. **(A)**. Deaths; **(B)**. Disability-adjusted life-years.

Supplementary Figure 2 Type 2 diabetes mellitus burden attributable to particulate matter population among different age subgroups from 1990 to 2019. **(A)**. Type 2 diabetes mellitus deaths attributable to APMP; **(B)**. Type 2 diabetes mellitus DALYs attributable to APMP; **(C)**. Type 2 diabetes mellitus deaths attributable to HAP; **(D)**. Type 2 diabetes mellitus DALYs attributable to HAP. DALY: disability-adjusted life-year; APMP ambient particulate matter pollution; HAP household air pollution.

Supplementary Figure 3 Type 2 diabetes mellitus ASDR attributable to particulate matter pollution in 2019 and their percentage changes from 1990 to 2019 across 21 GBD regions among different genders. **(A)**. Type 2 diabetes mellitus ASDR attributable to APMP; **(B)**. Percentage changes in type 2 diabetes mellitus ASDR attributable to APMP from 1990 to 2019; **(C)**. Type 2 diabetes mellitus ASDR attributable to HAP; **(D)**. Percentage changes in type 2 diabetes mellitus ASDR attributable to HAP from 1990 to 2019. ASDR age-standardized death rate; APMP ambient particulate matter pollution; HAP household air pollution.

Supplementary Figure 4 Type 2 diabetes mellitus ASDR attributable to particulate matter pollution among 204 countries and territories in 2019.**(A)**. APMP; **(B)**. HAP. APMP ambient particulate matter pollution; HAP household air pollution.

Supplementary Table 1 Global and regional age-standardized burden of type 2 diabetes mellitus burdens attributable to particulate matter pollution in 1990 and 2019.

Supplementary Table 2 Annualized rate of change in age-standardized rates for type 2 diabetes mellitus attributable to particulate matter pollution for both sexes in 204 countries and territories, 1990 to 2019.
