# Supplementary figures and images for: Estimates of Type 2 Diabetes Mellitus Burden Attributable to Particulate Matter Pollution and Its 30-Year Change Patterns: A Systematic Analysis of Data From the Global Burden of Disease Study 2019
Source: Front Endocrinol (Lausanne). 2021 Aug 13;12:689079. doi: 10.3389/fendo.2021.689079 (PMC8414895; doi:10.3389/fendo.2021.689079)

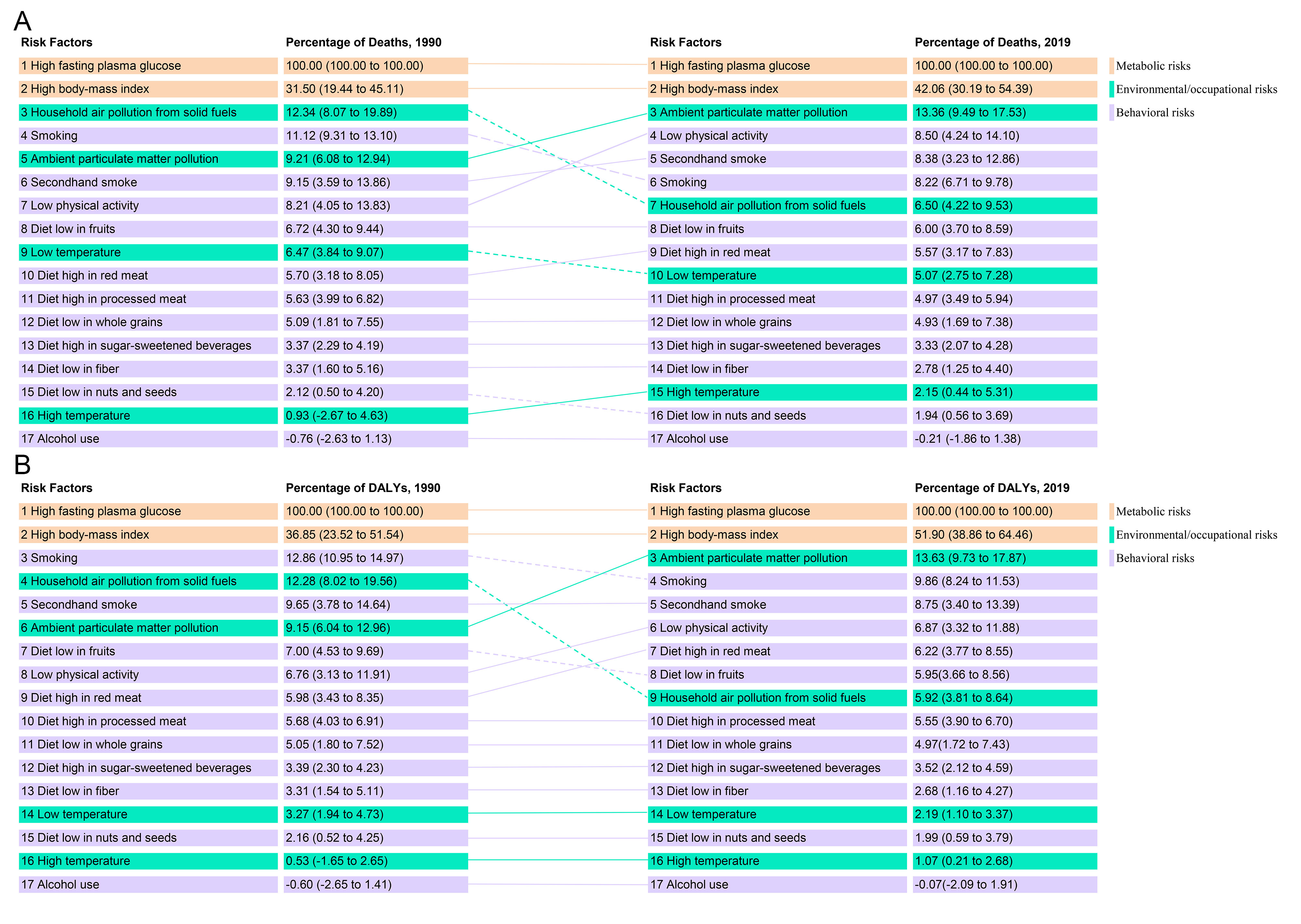

Supplement: Supplementary Figure 1 — Ranking changes of all risk factors for type 2 diabetes mellitus from 1990 to 2019 for both sexes combined for all ages. (A). Deaths; (B). Disability-adjusted life-years. [file Image_1.jpeg]

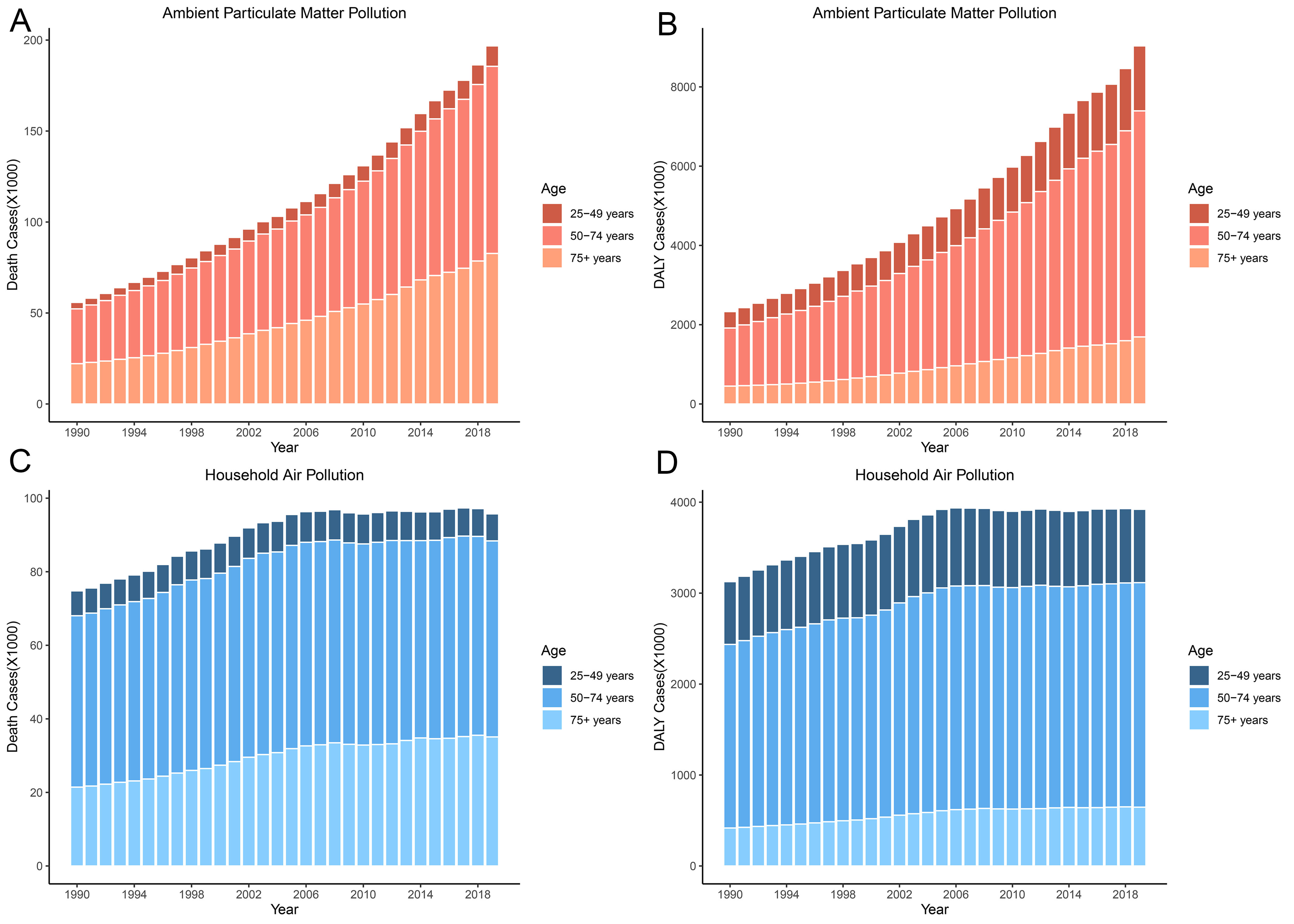

Supplement: Supplementary Figure 2 — Type 2 diabetes mellitus burden attributable to particulate matter population among different age subgroups from 1990 to 2019. (A). Type 2 diabetes mellitus deaths attributable to APMP; (B). Type 2 diabetes mellitus DALYs attributable to APMP; (C). Type 2 diabetes mellitus deaths attributable to HAP; (D). Type 2 diabetes mellitus DALYs attributable to HAP. DALY: disability-adjusted life-year; APMP ambient particulate matter pollution; HAP household air pollution. [file Image_2.jpeg]

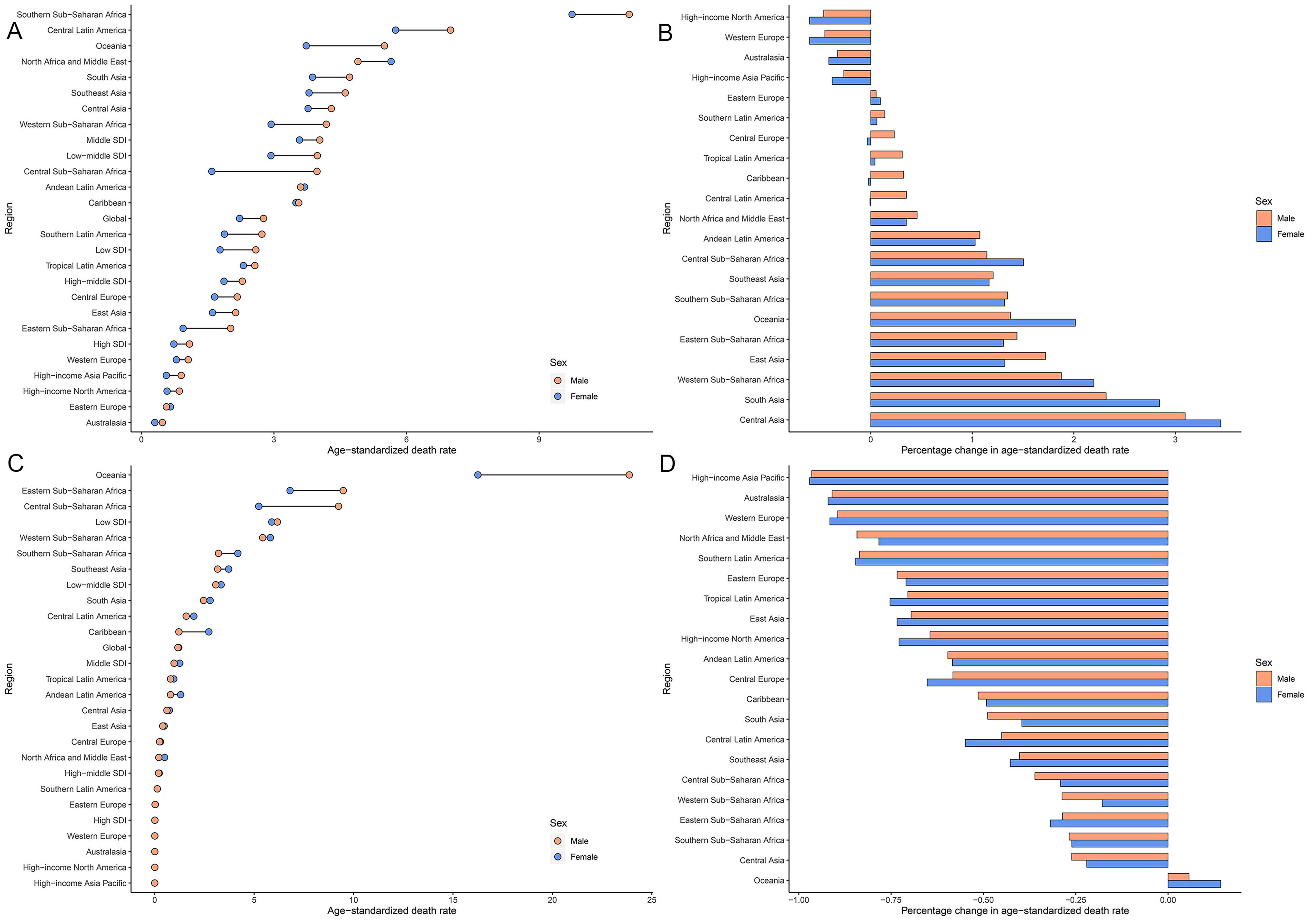

Supplement: Supplementary Figure 3 — Type 2 diabetes mellitus ASDR attributable to particulate matter pollution in 2019 and their percentage changes from 1990 to 2019 across 21 GBD regions among different genders. (A). Type 2 diabetes mellitus ASDR attributable to APMP; (B). Percentage changes in type 2 diabetes mellitus ASDR attributable to APMP from 1990 to 2019; (C). Type 2 diabetes mellitus ASDR attributable to HAP; (D). Percentage changes in type 2 diabetes mellitus ASDR attributable to HAP from 1990 to 2019. ASDR age-standardized death rate; APMP ambient particulate matter pollution; HAP household air pollution. [file Image_3.jpeg]

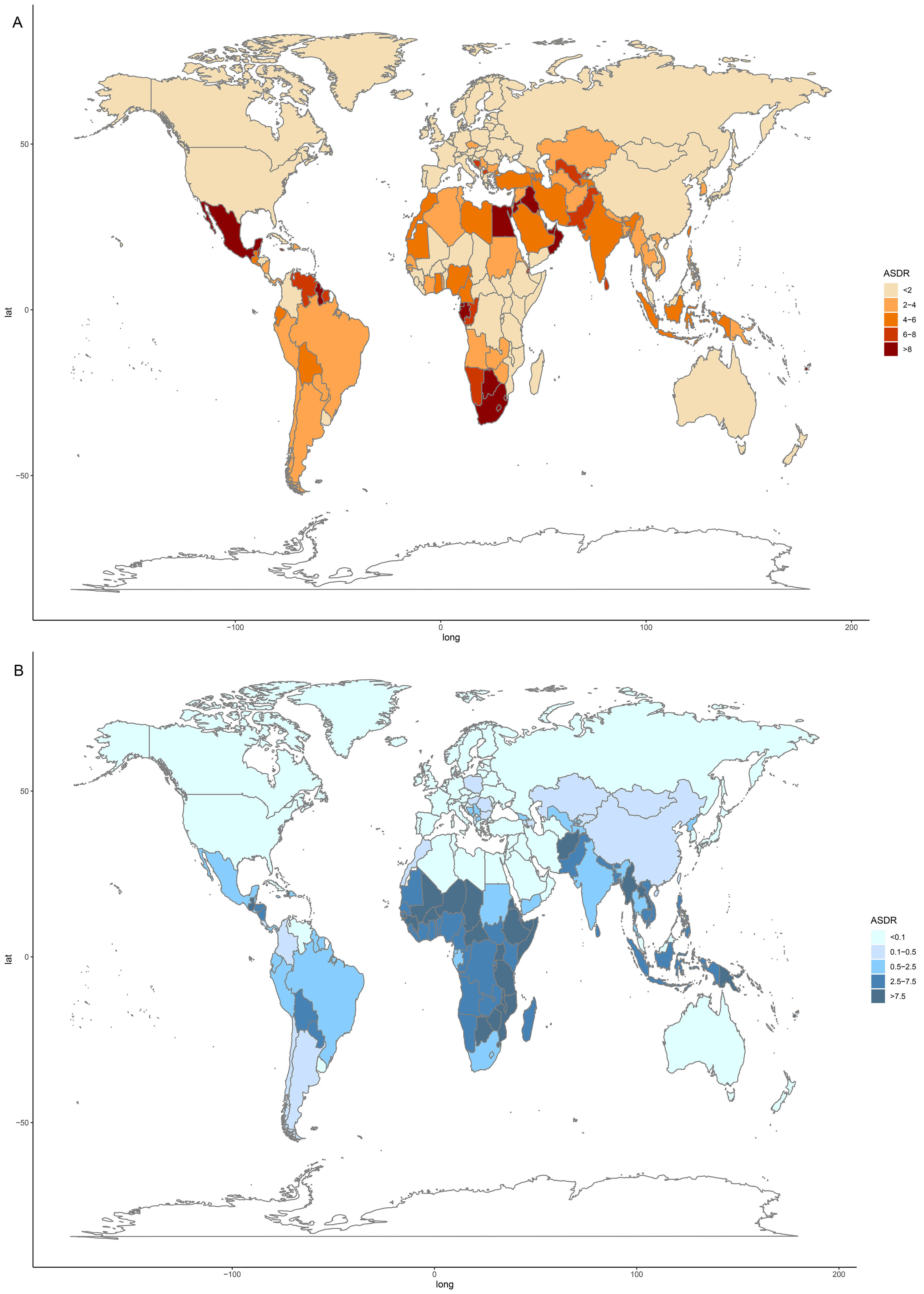

Supplement: Supplementary Figure 4 — Type 2 diabetes mellitus ASDR attributable to particulate matter pollution among 204 countries and territories in 2019.(A). APMP; (B). HAP. APMP ambient particulate matter pollution; HAP household air pollution. [file Image_4.jpeg]
