# Supplementary material for: Estimates of Type 2 Diabetes Mellitus Burden Attributable to Particulate Matter Pollution and Its 30-Year Change Patterns: A Systematic Analysis of Data From the Global Burden of Disease Study 2019
Source: Front Endocrinol (Lausanne). 2021 Aug 13;12:689079. doi: 10.3389/fendo.2021.689079 (PMC8414895; doi:10.3389/fendo.2021.689079)
Supplement: Supplementary Table 1 — Global and regional age-standardized burden of type 2 diabetes mellitus burdens attributable to particulate matter pollution in 1990 and 2019. [file Table_1.docx]

**Supplementary Table 1 Global and regional age-standardized burden of type 2 diabetes mellitus burdens attributable to particulate matter pollution in 1990 and 2019.**

|  | **APMP** | | | | **HAP** | | | |
| --- | --- | --- | --- | --- | --- | --- | --- | --- |
|  | **ASDR (95% UI)** | | **Age-standardized DALY rate (95% UI)** | | **ASDR (95% UI)** | | **Age-standardized DALY rate (95% UI)** | |
|  | **1990** | **2019** | **1990** | **2019** | **1990** | **2019** | **1990** | **2019** |
| **Global** | 1.57(1.04 to 2.21) | 2.47(1.71 to 3.24) | 58.36(36.59 to 83.10) | 108.98(74.06 to 147.23) | 1.98(1.29 to 3.18) | 1.19(0.75 to 1.71) | 75.85(48.12 to 120.41) | 47.14(29.24 to 70.37) |
| Male | 1.62(1.06 to 2.28) | 2.76(1.94 to 3.66) | 62.55(39.08 to 89.38) | 122.21(84.12 to 165.91) | 2.06(1.33 to 3.36) | 1.16(0.71 to 1.75) | 77.02(48.5 to 122.82) | 45.62(26.38 to 69.7) |
| Female | 1.52(1.01 to 2.14) | 2.22(1.54 to 2.91) | 54.42(34.32 to 77.87) | 96.89(65.3 to 132.54) | 1.93(1.26 to 3.15) | 1.21(0.78 to 1.79) | 75.34(47.59 to 123.95) | 48.56(30.54 to 72.9) |
| **SDI rank** |  |  |  |  |  |  |  |  |
| High SDI | 1.57(0.93 to 2.31) | 0.90(0.56 to 1.33) | 64.16(36.52 to 97.13) | 58.63(33.80 to 91.13) | 0.07(0.04 to 0.12) | 0.01(0.00 to 0.02) | 3.08(1.57 to 5.24) | 0.38(0.10 to 1.04) |
| High-middle SDI | 1.72(1.16 to 2.37) | 2.05(1.46 to 2.66) | 67.22(43.63 to 95.71) | 100.16(68.29 to 137.91) | 0.80(0.50 to 1.18) | 0.21(0.09 to 0.39) | 36.56(22.20 to 55.28) | 10.26(4.21 to 19.84) |
| Middle SDI | 1.83(1.14 to 2.71) | 3.79(2.70 to 4.86) | 65.15(38.66 to 97.65) | 150.14(103.58 to 200.72) | 2.82(1.89 to 4.00) | 1.13(0.63 to 1.82) | 102.28(67.05 to 146.89) | 40.92(21.83 to 66.55) |
| Low-middle SDI | 0.94(0.41 to 1.75) | 3.41(2.18 to 4.79) | 31.66(13.48 to 58.69) | 125.41(79.54 to 177.60) | 4.95(3.18 to 8.35) | 3.21(2.04 to 4.69) | 163.44(102.00 to 284.64) | 114.69(71.29 to 169.81) |
| Low SDI | 0.77(0.22 to 1.69) | 2.16(1.12 to 3.46) | 24.36(7.19 to 53.98) | 76.33(40.76 to 121.87) | 8.15(4.91 to 16.41) | 6.02(3.93 to 9.45) | 242.40(144.62 to 489.71) | 195.22(123.29 to 305.67) |
| **21 GBD regions** |  |  |  |  |  |  |  |  |
| Andean Latin America | 1.78(0.76 to 3.18) | 3.66(2.36 to 5.10) | 60.28(25.99 to 104.51) | 133.84(85.15 to 184.64) | 2.57(1.56 to 3.74) | 1.06(0.53 to 1.75) | 85.38(51.72 to 124.88) | 37.32(18.17 to 62.53) |
| Australasia | 0.59(0.05 to 1.44) | 0.38(0.09 to 0.78) | 19.71(1.82 to 48.04) | 18.31(4.08 to 38.78) | 0.04(0.00 to 0.10) | 0.00(0.00 to 0.01) | 1.15(0.15 to 3.45) | 0.14(0.02 to 0.45) |
| Caribbean | 3.16(1.26 to 5.26) | 3.54(1.96 to 5.53) | 113.77(44.48 to 197.75) | 150.91(81.57 to 235.09) | 3.99(2.67 to 6.00) | 2.01(1.26 to 3.05) | 130.46(87.18 to 199.74) | 83.34(52.41 to 124.99) |
| Central Asia | 0.93(0.43 to 1.52) | 4.01(2.69 to 5.52) | 47.13(20.67 to 81.71) | 169.40(110.80 to 237.02) | 0.91(0.54 to 1.32) | 0.69(0.33 to 1.27) | 46.09(25.54 to 68.88) | 29.83(14.03 to 55.29) |
| Central Europe | 1.75(0.97 to 2.52) | 1.90(1.27 to 2.52) | 86.74(45.74 to 131.04) | 115.15(72.83 to 161.56) | 0.73(0.38 to 1.22) | 0.27(0.10 to 0.58) | 35.75(18.31 to 58.33) | 15.09(5.30 to 32.72) |
| Central Latin America | 5.51(2.95 to 8.35) | 6.34(4.33 to 8.54) | 192.70(102.55 to 297.19) | 246.53(161.84 to 339.83) | 3.66(2.25 to 5.36) | 1.79(1.03 to 2.82) | 138.49(86.44 to 201.62) | 69.91(40.88 to 110.94) |
| Central Sub-Saharan Africa | 1.20(0.33 to 2.79) | 2.56(1.28 to 4.19) | 33.93(9.52 to 78.45) | 81.80(41.84 to 137.88) | 10.67(6.55 to 19.62) | 6.88(4.38 to 10.41) | 309.30(188.90 to 566.37) | 228.45(145.87 to 351.07) |
| East Asia | 0.72(0.33 to 1.25) | 1.80(1.28 to 2.38) | 34.21(15.20 to 61.18) | 90.83(60.49 to 124.49) | 1.55(0.97 to 2.29) | 0.44(0.23 to 0.75) | 76.61(46.85 to 114.76) | 22.09(10.85 to 38.37) |
| Eastern Europe | 0.58(0.28 to 0.91) | 0.63(0.33 to 0.97) | 39.64(18.13 to 65.90) | 39.97(20.21 to 63.77) | 0.09(0.04 to 0.17) | 0.03(0.01 to 0.07) | 6.16(2.64 to 11.54) | 1.76(0.52 to 4.46) |
| Eastern Sub-Saharan Africa | 0.61(0.18 to 1.45) | 1.43(0.70 to 2.50) | 16.31(4.70 to 38.67) | 40.18(19.83 to 70.89) | 11.58(7.08 to 23.35) | 8.01(5.31 to 12.28) | 314.87(187.87 to 640.36) | 223.53(147.21 to 346.43) |
| High-income Asia Pacific | 1.06(0.44 to 1.70) | 0.73(0.49 to 0.97) | 51.23(19.44 to 84.09) | 59.19(35.83 to 88.06) | 0.03(0.01 to 0.07) | 0.00(0.00 to 0.00) | 1.29(0.42 to 3.13) | 0.09(0.02 to 0.28) |
| High-income North America | 1.52(0.56 to 2.67) | 0.71(0.32 to 1.26) | 68.28(23.85 to 121.39) | 43.06(18.72 to 77.52) | 0.01(0.00 to 0.02) | 0.00(0.00 to 0.01) | 0.31(0.08 to 0.81) | 0.13(0.02 to 0.43) |
| North Africa and Middle East | 3.79(2.65 to 5.06) | 5.28(3.80 to 6.76) | 121.82(83.54 to 162.60) | 220.55(156.88 to 293.20) | 1.80(1.07 to 2.90) | 0.35(0.20 to 0.57) | 61.03(36.25 to 98.94) | 16.02(9.46 to 24.94) |
| Oceania | 1.77(0.47 to 4.79) | 4.61(1.34 to 10.67) | 50.99(13.84 to 140.82) | 133.27(39.87 to 306.72) | 18.32(12.04 to 28.74) | 20.01(13.01 to 28.87) | 545.96(356.17 to 868.62) | 630.83(409.93 to 914.27) |
| South Asia | 1.22(0.48 to 2.32) | 4.27(2.85 to 5.79) | 41.80(17.40 to 79.64) | 160.25(106.33 to 220.58) | 4.71(2.90 to 8.21) | 2.63(1.61 to 4.02) | 153.26(92.15 to 268.17) | 95.88(56.92 to 147.49) |
| Southeast Asia | 1.90(0.89 to 3.32) | 4.18(2.71 to 5.89) | 60.56(27.77 to 106.85) | 141.80(90.88 to 203.20) | 5.97(3.91 to 8.61) | 3.50(2.13 to 5.11) | 180.09(117.98 to 264.67) | 112.92(67.97 to 167.48) |
| Southern Latin America | 2.05(0.74 to 3.60) | 2.25(1.43 to 3.19) | 64.25(24.49 to 113.46) | 95.10(58.78 to 139.32) | 0.82(0.43 to 1.38) | 0.13(0.05 to 0.29) | 26.09(12.96 to 44.83) | 5.47(1.83 to 12.48) |
| Southern Sub-Saharan Africa | 4.42(2.89 to 6.16) | 10.29(7.09 to 13.55) | 124.34(80.18 to 172.02) | 280.44(189.36 to 372.20) | 5.14(3.46 to 7.33) | 3.84(2.31 to 5.80) | 150.21(100.07 to 210.87) | 106.84(63.47 to 160.61) |
| Tropical Latin America | 2.11(0.87 to 3.75) | 2.44(1.47 to 3.70) | 75.10(31.35 to 132.36) | 90.72(52.41 to 138.96) | 3.36(2.14 to 4.79) | 0.89(0.43 to 1.57) | 107.92(68.59 to 153.97) | 29.96(13.64 to 54.12) |
| Western Europe | 2.00(0.98 to 3.07) | 0.92(0.56 to 1.36) | 69.06(32.73 to 109.82) | 53.11(29.90 to 83.57) | 0.05(0.02 to 0.11) | 0.00(0.00 to 0.01) | 1.74(0.65 to 3.85) | 0.25(0.07 to 0.69) |
| Western Sub-Saharan Africa | 1.17(0.43 to 2.35) | 3.52(2.02 to 5.41) | 30.78(11.07 to 63.52) | 94.45(54.13 to 145.88) | 7.33(4.24 to 14.46) | 5.63(3.45 to 8.86) | 197.99(115.68 to 387.77) | 160.49(97.63 to 255.17) |

Abbreviations: APMP ambient particulate matter pollution; HAP household air pollution; ASDR age-standardized death rate; DALY disability-adjusted life year; SDI socio-demographic index; GBD global burden of disease; UI uncertainty interval.
