# Supplementary material for: Estimates of Type 2 Diabetes Mellitus Burden Attributable to Particulate Matter Pollution and Its 30-Year Change Patterns: A Systematic Analysis of Data From the Global Burden of Disease Study 2019
Source: Front Endocrinol (Lausanne). 2021 Aug 13;12:689079. doi: 10.3389/fendo.2021.689079 (PMC8414895; doi:10.3389/fendo.2021.689079)
Supplement: Supplementary Table 2 — Annualized rate of change in age-standardized rates for type 2 diabetes mellitus attributable to particulate matter pollution for both sexes in 204 countries and territories, 1990 to 2019. [file Table_2.docx]

**Supplementary Table 2 Annualized rate of change in age-standardized rates for type 2 diabetes mellitus attributable to particulate matter pollution for both sexes in 204 countries and territories, 1990 to 2019.**

| **Location** | **APMP** | | **HAP** | |
| --- | --- | --- | --- | --- |
|  | **Percentage change in ASDR, 1990-2019 (95% UI)** | **Percentage change in age-standardized DALY rate, 1990-2019 (95% UI)** | **Percentage change in ASDR, 1990-2019 (95% UI)** | **Percentage change in age-standardized DALY rate, 1990-2019 (95% UI)** |
| Afghanistan | 2.87(1.02 to 9.19) | 3.29(1.32 to 10.49) | 0.03(-0.34 to 0.53) | 0.13(-0.23 to 0.57) |
| Albania | 0.42(-0.17 to 1.84) | 1.26(0.38 to 3.41) | -0.68(-0.84 to -0.48) | -0.49(-0.73 to -0.22) |
| Algeria | 0.39(0.02 to 1.01) | 0.93(0.54 to 1.57) | -0.98(-0.99 to -0.96) | -0.98(-0.99 to -0.95) |
| American Samoa | 0.47(-0.51 to 3.53) | 0.65(-0.45 to 3.91) | -0.54(-0.85 to -0.12) | -0.49(-0.84 to -0.03) |
| Andorra | -0.53(-0.76 to 0.14) | -0.17(-0.56 to 1) | -0.85(-0.95 to -0.66) | -0.74(-0.89 to -0.43) |
| Angola | 3.8(1.44 to 11.99) | 3.97(1.61 to 12.22) | -0.5(-0.71 to -0.18) | -0.48(-0.71 to -0.2) |
| Antigua and Barbuda | 0.13(-0.35 to 1.79) | 0.22(-0.29 to 2.07) | -0.84(-0.93 to -0.64) | -0.83(-0.93 to -0.61) |
| Argentina | -0.02(-0.45 to 1.33) | 0.29(-0.26 to 2.05) | -0.85(-0.93 to -0.72) | -0.79(-0.9 to -0.64) |
| Armenia | 1.34(0.63 to 3.13) | 1.3(0.63 to 3.02) | -0.86(-0.95 to -0.71) | -0.87(-0.95 to -0.72) |
| Australia | -0.35(-0.8 to 2.68) | -0.05(-0.71 to 4.34) | -0.92(-0.98 to -0.71) | -0.88(-0.97 to -0.56) |
| Austria | -0.4(-0.59 to 0) | -0.15(-0.43 to 0.42) | -0.79(-0.92 to -0.56) | -0.7(-0.88 to -0.37) |
| Azerbaijan | 2.87(1.24 to 6.94) | 2.49(1.07 to 6.06) | -0.69(-0.86 to -0.43) | -0.72(-0.87 to -0.5) |
| Bahamas | -0.19(-0.6 to 0.92) | 0.03(-0.48 to 1.37) | -0.87(-0.94 to -0.72) | -0.83(-0.93 to -0.64) |
| Bahrain | 0.77(0.32 to 1.24) | 0.69(0.37 to 1.02) | -0.95(-0.98 to -0.87) | -0.95(-0.98 to -0.88) |
| Bangladesh | 2.55(0.78 to 9.57) | 2.69(0.93 to 9.7) | -0.38(-0.62 to -0.09) | -0.36(-0.6 to -0.1) |
| Barbados | -0.09(-0.44 to 0.8) | 0.02(-0.37 to 1.05) | -0.82(-0.92 to -0.57) | -0.79(-0.91 to -0.53) |
| Belarus | -0.56(-0.7 to -0.26) | -0.21(-0.45 to 0.27) | -0.94(-0.98 to -0.86) | -0.9(-0.96 to -0.77) |
| Belgium | -0.61(-0.74 to -0.34) | -0.21(-0.48 to 0.34) | -0.91(-0.97 to -0.8) | -0.81(-0.93 to -0.58) |
| Belize | 1.67(0.45 to 6.86) | 1.99(0.63 to 7.85) | -0.56(-0.77 to -0.24) | -0.5(-0.73 to -0.17) |
| Benin | 1.86(0.86 to 4.59) | 2.09(1.06 to 4.94) | 0.02(-0.28 to 0.41) | 0.09(-0.21 to 0.44) |
| Bermuda | -0.62(-0.91 to 0.54) | -0.42(-0.86 to 1.3) | -0.92(-0.98 to -0.81) | -0.87(-0.97 to -0.71) |
| Bhutan | 7.12(2.75 to 26.4) | 6.65(2.65 to 24.28) | -0.16(-0.51 to 0.33) | -0.2(-0.53 to 0.19) |
| Bolivia (Plurinational State of) | 0.68(-0.09 to 2.69) | 0.69(-0.03 to 2.71) | -0.42(-0.65 to -0.08) | -0.41(-0.64 to -0.1) |
| Bosnia and Herzegovina | 3.77(1.88 to 8.03) | 2.88(1.51 to 6.25) | -0.08(-0.58 to 0.59) | -0.25(-0.64 to 0.27) |
| Botswana | 3.42(1.66 to 6.99) | 3.42(1.75 to 6.86) | -0.33(-0.63 to 0.05) | -0.33(-0.62 to 0.04) |
| Brazil | 0.13(-0.38 to 1.48) | 0.19(-0.35 to 1.57) | -0.75(-0.87 to -0.6) | -0.74(-0.87 to -0.58) |
| Brunei Darussalam | -0.32(-0.78 to 0.88) | -0.2(-0.74 to 1.16) | -0.97(-0.99 to -0.93) | -0.96(-0.99 to -0.92) |
| Bulgaria | -0.09(-0.39 to 0.59) | 0.14(-0.19 to 0.98) | -0.53(-0.79 to -0.13) | -0.42(-0.73 to 0.08) |
| Burkina Faso | 1(0.41 to 2.69) | 1.25(0.6 to 3.11) | -0.16(-0.38 to 0.14) | -0.05(-0.29 to 0.23) |
| Burundi | 0.14(-0.16 to 0.74) | 0.21(-0.09 to 0.85) | -0.27(-0.46 to -0.01) | -0.24(-0.41 to 0) |
| Cabo Verde | 13.58(6.7 to 33.64) | 8.89(4.61 to 21.85) | 0.01(-0.49 to 0.7) | -0.33(-0.64 to 0.07) |
| Cambodia | 1.49(0.22 to 5.75) | 2.11(0.55 to 7.59) | -0.25(-0.52 to 0.03) | -0.08(-0.39 to 0.25) |
| Cameroon | 1.85(0.81 to 4.42) | 2(0.94 to 4.6) | -0.13(-0.44 to 0.3) | -0.09(-0.39 to 0.28) |
| Canada | -0.55(-0.81 to 0.25) | -0.27(-0.68 to 0.99) | -0.94(-0.98 to -0.87) | -0.9(-0.96 to -0.79) |
| Central African Republic | 0.54(0.13 to 1.49) | 0.68(0.26 to 1.63) | -0.1(-0.34 to 0.22) | -0.03(-0.26 to 0.26) |
| Chad | 1.71(0.89 to 4.18) | 1.83(0.99 to 4.34) | 0.14(-0.17 to 0.57) | 0.19(-0.13 to 0.56) |
| Chile | 0.49(-0.02 to 1.97) | 0.83(0.21 to 2.6) | -0.84(-0.93 to -0.71) | -0.81(-0.92 to -0.63) |
| China | 1.65(0.57 to 4.37) | 1.75(0.68 to 4.49) | -0.72(-0.85 to -0.55) | -0.72(-0.84 to -0.55) |
| Colombia | 0.03(-0.39 to 1.1) | 0.48(-0.08 to 1.88) | -0.84(-0.92 to -0.72) | -0.76(-0.88 to -0.62) |
| Comoros | 1.44(0.48 to 3.77) | 1.59(0.6 to 4.02) | -0.27(-0.51 to 0.12) | -0.23(-0.47 to 0.18) |
| Congo | 1.59(0.46 to 4.69) | 1.71(0.58 to 4.81) | -0.54(-0.73 to -0.32) | -0.52(-0.71 to -0.3) |
| Cook Islands | 0.12(-0.68 to 2.03) | 0.28(-0.64 to 2.47) | -0.76(-0.94 to -0.49) | -0.73(-0.93 to -0.43) |
| Costa Rica | 0.39(-0.27 to 2.22) | 1.07(0.16 to 3.78) | -0.88(-0.94 to -0.79) | -0.82(-0.91 to -0.68) |
| Croatia | -0.09(-0.38 to 0.63) | 0.18(-0.14 to 0.98) | -0.76(-0.9 to -0.52) | -0.69(-0.87 to -0.36) |
| Cuba | -0.49(-0.71 to 0.11) | -0.05(-0.43 to 1.05) | -0.9(-0.95 to -0.8) | -0.8(-0.91 to -0.63) |
| Cyprus | -0.59(-0.74 to -0.17) | -0.4(-0.61 to 0.19) | -0.97(-0.99 to -0.93) | -0.96(-0.98 to -0.9) |
| Czechia | -0.04(-0.31 to 0.55) | 0.23(-0.09 to 0.95) | -0.67(-0.87 to -0.26) | -0.57(-0.83 to -0.05) |
| Cote d'Ivoire | 1.42(0.59 to 3.51) | 1.61(0.75 to 3.75) | -0.12(-0.39 to 0.23) | -0.05(-0.34 to 0.26) |
| Democratic People's Republic of Korea | 0.86(0.07 to 2.78) | 1.35(0.36 to 3.68) | -0.34(-0.55 to -0.07) | -0.17(-0.4 to 0.09) |
| Democratic Republic of the Congo | 0.39(-0.09 to 1.44) | 0.68(0.14 to 1.91) | -0.29(-0.49 to -0.06) | -0.16(-0.37 to 0.07) |
| Denmark | -0.32(-0.6 to 0.36) | -0.2(-0.53 to 0.58) | -0.74(-0.9 to -0.41) | -0.68(-0.88 to -0.31) |
| Djibouti | 3.63(1.55 to 10.31) | 3.66(1.61 to 10.29) | -0.59(-0.8 to -0.28) | -0.59(-0.8 to -0.29) |
| Dominica | 0.72(-0.09 to 3.09) | 1.1(0.13 to 4.02) | -0.85(-0.94 to -0.72) | -0.82(-0.92 to -0.66) |
| Dominican Republic | 3.56(1.27 to 12.4) | 4.02(1.53 to 13.62) | -0.7(-0.86 to -0.46) | -0.67(-0.84 to -0.41) |
| Ecuador | 1.68(0.56 to 4.82) | 1.62(0.57 to 4.56) | -0.72(-0.87 to -0.5) | -0.73(-0.87 to -0.52) |
| Egypt | 0.68(0.25 to 1.25) | 0.98(0.6 to 1.48) | -0.99(-1 to -0.98) | -0.99(-1 to -0.98) |
| El Salvador | 5.04(2.07 to 14.19) | 4.63(1.99 to 12.67) | -0.47(-0.71 to -0.13) | -0.5(-0.71 to -0.23) |
| Equatorial Guinea | 10.17(3.3 to 50.53) | 9.28(3.1 to 45.45) | -0.82(-0.95 to -0.56) | -0.84(-0.95 to -0.6) |
| Eritrea | 2.18(0.88 to 6.5) | 2.08(0.89 to 5.9) | -0.15(-0.46 to 0.32) | -0.18(-0.46 to 0.18) |
| Estonia | -0.27(-0.72 to 0.84) | -0.22(-0.7 to 0.83) | -0.72(-0.94 to -0.31) | -0.7(-0.93 to -0.31) |
| Eswatini | 2.92(1.39 to 6.07) | 2.88(1.42 to 5.89) | -0.01(-0.36 to 0.46) | -0.02(-0.35 to 0.42) |
| Ethiopia | 1.07(0.2 to 4.27) | 0.96(0.14 to 3.88) | -0.53(-0.71 to -0.31) | -0.56(-0.71 to -0.36) |
| Fiji | 3.5(0.88 to 12.95) | 3.38(0.86 to 12.4) | -0.3(-0.64 to 0.16) | -0.33(-0.65 to 0.1) |
| Finland | -0.79(-0.96 to -0.02) | -0.49(-0.9 to 1.36) | -0.94(-0.99 to -0.83) | -0.84(-0.97 to -0.54) |
| France | -0.38(-0.6 to 0.14) | -0.16(-0.47 to 0.5) | -0.83(-0.93 to -0.63) | -0.77(-0.9 to -0.51) |
| Gabon | 2.27(0.84 to 5.94) | 2.34(0.94 to 6.2) | -0.9(-0.96 to -0.8) | -0.9(-0.96 to -0.8) |
| Gambia | 2.45(1.06 to 6.77) | 2.53(1.16 to 6.64) | 0.12(-0.27 to 0.69) | 0.15(-0.23 to 0.65) |
| Georgia | 1.69(0.65 to 4.01) | 1.7(0.69 to 3.85) | 0.21(-0.25 to 0.82) | 0.21(-0.24 to 0.74) |
| Germany | -0.59(-0.72 to -0.3) | -0.3(-0.55 to 0.21) | -0.79(-0.91 to -0.56) | -0.64(-0.86 to -0.27) |
| Ghana | 2.66(1.24 to 5.99) | 2.81(1.43 to 6.18) | -0.22(-0.53 to 0.17) | -0.18(-0.5 to 0.19) |
| Greece | -0.45(-0.63 to -0.03) | 0.09(-0.28 to 0.93) | -0.92(-0.96 to -0.82) | -0.83(-0.93 to -0.63) |
| Greenland | -0.46(-0.91 to 1.01) | -0.01(-0.83 to 2.72) | -0.91(-0.99 to -0.75) | -0.82(-0.97 to -0.55) |
| Grenada | 1.25(0.26 to 5.54) | 1.41(0.35 to 6.14) | -0.92(-0.97 to -0.84) | -0.92(-0.97 to -0.83) |
| Guam | -0.43(-0.82 to 2.3) | -0.06(-0.69 to 4.36) | -0.66(-0.88 to -0.09) | -0.44(-0.8 to 0.45) |
| Guatemala | 7.35(3.03 to 20.35) | 5.58(2.22 to 15.59) | 1.2(0.31 to 2.2) | 0.73(0.07 to 1.45) |
| Guinea | 1.58(0.69 to 3.78) | 1.75(0.87 to 4.02) | 0.11(-0.23 to 0.59) | 0.18(-0.17 to 0.61) |
| Guinea-Bissau | 1.28(0.47 to 3.65) | 1.35(0.56 to 3.71) | -0.03(-0.33 to 0.39) | -0.02(-0.31 to 0.36) |
| Guyana | 0.77(-0.05 to 4.36) | 0.86(0.01 to 4.5) | -0.72(-0.85 to -0.5) | -0.7(-0.84 to -0.49) |
| Haiti | 0.68(-0.17 to 3.37) | 0.97(0.02 to 4.04) | -0.33(-0.54 to -0.06) | -0.22(-0.42 to 0.02) |
| Honduras | 2.44(0.75 to 7.63) | 2.21(0.69 to 6.76) | 0.07(-0.26 to 0.44) | 0(-0.29 to 0.25) |
| Hungary | -0.02(-0.33 to 0.73) | 0.24(-0.12 to 1.12) | -0.58(-0.82 to -0.17) | -0.46(-0.77 to 0.02) |
| Iceland | -0.52(-0.89 to 2.07) | -0.08(-0.79 to 4.81) | -0.9(-0.98 to -0.6) | -0.8(-0.96 to -0.2) |
| India | 2.17(0.92 to 5.38) | 2.64(1.27 to 6.28) | -0.5(-0.67 to -0.28) | -0.42(-0.61 to -0.18) |
| Indonesia | 1.98(0.69 to 5.11) | 1.9(0.64 to 4.84) | -0.31(-0.58 to 0) | -0.32(-0.58 to -0.03) |
| Iran (Islamic Republic of) | 0.94(0.57 to 1.37) | 1.1(0.88 to 1.38) | -0.97(-0.99 to -0.94) | -0.97(-0.99 to -0.94) |
| Iraq | 0.27(-0.1 to 0.85) | 0.44(0.09 to 1.02) | -0.99(-1 to -0.98) | -0.99(-1 to -0.98) |
| Ireland | -0.66(-0.84 to -0.13) | -0.23(-0.65 to 0.98) | -0.93(-0.98 to -0.84) | -0.84(-0.95 to -0.62) |
| Israel | 0.16(-0.14 to 0.87) | 0.26(-0.06 to 1.03) | -0.73(-0.89 to -0.41) | -0.71(-0.88 to -0.36) |
| Italy | -0.51(-0.65 to -0.22) | -0.21(-0.45 to 0.26) | -0.9(-0.96 to -0.78) | -0.84(-0.93 to -0.65) |
| Jamaica | 1.96(0.51 to 6.24) | 2.12(0.59 to 6.44) | -0.77(-0.89 to -0.58) | -0.75(-0.88 to -0.57) |
| Japan | -0.65(-0.79 to -0.09) | 0.03(-0.39 to 1.72) | -0.96(-0.98 to -0.9) | -0.87(-0.95 to -0.71) |
| Jordan | -0.33(-0.47 to -0.15) | -0.16(-0.3 to 0.01) | -0.99(-1 to -0.98) | -0.99(-1 to -0.97) |
| Kazakhstan | 2.49(1.05 to 6.09) | 1.78(0.65 to 4.58) | -0.45(-0.76 to 0.01) | -0.56(-0.8 to -0.21) |
| Kenya | 1.74(0.81 to 3.44) | 1.94(0.97 to 3.75) | -0.14(-0.38 to 0.09) | -0.09(-0.34 to 0.12) |
| Kiribati | 1.21(0.03 to 4.15) | 1.21(0.06 to 4.11) | 0.03(-0.28 to 0.4) | 0.02(-0.27 to 0.37) |
| Kuwait | -0.35(-0.44 to -0.23) | 0.08(-0.05 to 0.19) | -0.98(-0.99 to -0.96) | -0.97(-0.99 to -0.94) |
| Kyrgyzstan | 0.99(0.13 to 3.23) | 1.4(0.35 to 3.95) | -0.47(-0.65 to -0.23) | -0.35(-0.57 to -0.1) |
| Lao People's Democratic Republic | 1.31(0.07 to 5.82) | 1.6(0.25 to 6.39) | -0.22(-0.51 to 0.2) | -0.13(-0.45 to 0.24) |
| Latvia | 0.23(-0.24 to 1.18) | 0.15(-0.25 to 0.95) | -0.47(-0.8 to 0.07) | -0.51(-0.81 to -0.05) |
| Lebanon | 0.01(-0.31 to 0.4) | 0.6(0.3 to 1.03) | -0.99(-1 to -0.97) | -0.98(-0.99 to -0.96) |
| Lesotho | 3.76(1.86 to 7.54) | 3.6(1.82 to 7.24) | 0.55(0.02 to 1.2) | 0.5(0 to 1.08) |
| Liberia | 1.26(0.46 to 3.09) | 1.47(0.71 to 3.33) | -0.04(-0.34 to 0.35) | 0.04(-0.24 to 0.36) |
| Libya | 0.86(0.26 to 2) | 1.37(0.86 to 2.32) | -0.99(-1 to -0.98) | -0.99(-1 to -0.97) |
| Lithuania | -0.27(-0.57 to 0.39) | -0.14(-0.48 to 0.66) | -0.82(-0.94 to -0.61) | -0.79(-0.93 to -0.54) |
| Luxembourg | -0.64(-0.79 to -0.22) | 0.19(-0.33 to 1.47) | -0.87(-0.95 to -0.72) | -0.58(-0.83 to -0.06) |
| Madagascar | 1.09(0.42 to 2.57) | 1.26(0.59 to 2.83) | -0.13(-0.39 to 0.19) | -0.07(-0.32 to 0.21) |
| Malawi | 0.98(0.3 to 3.03) | 1.22(0.46 to 3.35) | -0.23(-0.48 to 0.05) | -0.15(-0.42 to 0.12) |
| Malaysia | -0.57(-0.73 to -0.23) | -0.27(-0.53 to 0.25) | -0.96(-0.99 to -0.9) | -0.94(-0.98 to -0.83) |
| Maldives | 0.43(-0.41 to 3.44) | 0.95(-0.2 to 5.07) | -0.89(-0.95 to -0.79) | -0.85(-0.93 to -0.72) |
| Mali | 1.27(0.55 to 3.44) | 1.4(0.67 to 3.57) | -0.01(-0.29 to 0.39) | 0.04(-0.23 to 0.4) |
| Malta | -0.56(-0.73 to -0.09) | -0.22(-0.52 to 0.61) | -0.95(-0.98 to -0.89) | -0.91(-0.97 to -0.81) |
| Marshall Islands | 2.04(0.26 to 8) | 2.33(0.45 to 9.02) | -0.21(-0.51 to 0.26) | -0.14(-0.46 to 0.31) |
| Mauritania | 1.36(0.49 to 3.65) | 1.39(0.52 to 3.68) | -0.48(-0.68 to -0.23) | -0.47(-0.67 to -0.24) |
| Mauritius | 2.22(1.45 to 3.48) | 1.77(1.2 to 2.8) | -0.82(-0.93 to -0.62) | -0.84(-0.94 to -0.68) |
| Mexico | 0.07(-0.26 to 0.68) | 0.13(-0.22 to 0.79) | -0.56(-0.71 to -0.35) | -0.55(-0.7 to -0.35) |
| Micronesia (Federated States of) | 3.22(0.85 to 10.46) | 3.24(0.88 to 10.4) | -0.18(-0.57 to 0.32) | -0.18(-0.56 to 0.28) |
| Monaco | 0.27(-0.36 to 8.88) | 1.12(0.13 to 15.49) | -0.73(-0.89 to 0.3) | -0.53(-0.81 to 1.28) |
| Mongolia | 1.94(0.68 to 5.22) | 2.6(1.13 to 6.44) | -0.56(-0.76 to -0.29) | -0.46(-0.69 to -0.19) |
| Montenegro | 0.17(-0.18 to 0.83) | 0.38(0.02 to 1.13) | -0.26(-0.61 to 0.24) | -0.12(-0.52 to 0.42) |
| Morocco | 3.17(1.58 to 6.97) | 3.48(1.85 to 7.38) | -0.82(-0.92 to -0.66) | -0.81(-0.91 to -0.64) |
| Mozambique | 2.04(0.91 to 5.92) | 2.3(1.13 to 6.4) | 0(-0.34 to 0.48) | 0.07(-0.28 to 0.53) |
| Myanmar | 1.49(0.27 to 5.68) | 1.63(0.33 to 6.07) | -0.37(-0.6 to -0.07) | -0.33(-0.57 to -0.04) |
| Namibia | 1.58(0.67 to 3.41) | 1.59(0.71 to 3.32) | -0.4(-0.63 to -0.14) | -0.4(-0.62 to -0.15) |
| Nauru | 0.8(-0.58 to 6.27) | 0.85(-0.57 to 6.29) | -0.71(-0.94 to -0.32) | -0.7(-0.94 to -0.29) |
| Nepal | 4.78(1.8 to 16.98) | 4.71(1.92 to 16.6) | -0.08(-0.46 to 0.44) | -0.1(-0.43 to 0.29) |
| Netherlands | -0.65(-0.77 to -0.38) | -0.45(-0.65 to -0.03) | -0.92(-0.97 to -0.82) | -0.87(-0.95 to -0.72) |
| New Zealand | -0.44(-0.89 to 3.87) | -0.2(-0.85 to 5.63) | -0.87(-0.97 to -0.37) | -0.82(-0.96 to -0.13) |
| Nicaragua | 3.9(1.46 to 11.54) | 3.45(1.24 to 10.39) | -0.01(-0.34 to 0.35) | -0.11(-0.42 to 0.17) |
| Niger | 0.91(0.45 to 2.14) | 1.1(0.62 to 2.36) | 0.09(-0.15 to 0.45) | 0.2(-0.04 to 0.56) |
| Nigeria | 2.13(0.9 to 5.09) | 2.09(0.9 to 4.85) | -0.44(-0.62 to -0.15) | -0.42(-0.61 to -0.16) |
| Niue | 1.25(-0.42 to 5.49) | 1.39(-0.39 to 5.56) | -0.82(-0.96 to -0.58) | -0.81(-0.95 to -0.57) |
| North Macedonia | 1.11(0.48 to 2.32) | 1(0.48 to 2.03) | -0.28(-0.64 to 0.32) | -0.32(-0.65 to 0.17) |
| Northern Mariana Islands | 0.08(-0.56 to 2.21) | 0.21(-0.5 to 2.64) | -0.4(-0.73 to 0.05) | -0.32(-0.69 to 0.18) |
| Norway | -0.65(-0.88 to -0.03) | -0.5(-0.82 to 0.39) | -0.86(-0.96 to -0.66) | -0.81(-0.95 to -0.53) |
| Oman | 1.52(0.61 to 3.59) | 1.48(0.66 to 3.3) | -0.99(-1 to -0.97) | -0.99(-1 to -0.97) |
| Pakistan | 5.44(2.22 to 16.84) | 4.85(2 to 14.86) | 0.1(-0.34 to 0.73) | -0.02(-0.38 to 0.43) |
| Palau | 0.5(-0.92 to 119.75) | 0.58(-0.91 to 126.64) | -0.56(-0.97 to 18.38) | -0.53(-0.97 to 19.93) |
| Palestine | 1.66(0.61 to 4.29) | 1.83(0.77 to 4.46) | -0.96(-0.98 to -0.92) | -0.96(-0.98 to -0.91) |
| Panama | 1.32(0.2 to 4.38) | 1.34(0.29 to 4.31) | -0.61(-0.81 to -0.36) | -0.6(-0.8 to -0.37) |
| Papua New Guinea | 1.09(0.09 to 4.12) | 1.16(0.13 to 4.36) | 0.18(-0.13 to 0.6) | 0.23(-0.07 to 0.6) |
| Paraguay | 2.8(1 to 7.56) | 2.53(0.95 to 6.68) | -0.02(-0.4 to 0.44) | -0.09(-0.42 to 0.26) |
| Peru | 0.91(0.11 to 3.03) | 1.24(0.39 to 3.36) | -0.65(-0.81 to -0.41) | -0.58(-0.76 to -0.36) |
| Philippines | 0.26(-0.19 to 1.21) | 0.47(-0.05 to 1.51) | -0.28(-0.49 to 0) | -0.18(-0.4 to 0.08) |
| Poland | -0.06(-0.32 to 0.51) | 0.21(-0.11 to 0.91) | -0.75(-0.9 to -0.53) | -0.67(-0.87 to -0.39) |
| Portugal | -0.48(-0.75 to 0.53) | -0.25(-0.65 to 1.11) | -0.94(-0.98 to -0.87) | -0.92(-0.97 to -0.81) |
| Puerto Rico | -0.22(-0.83 to 103.54) | -0.02(-0.79 to 145.8) | -0.76(-0.94 to 11.34) | -0.69(-0.93 to 14.8) |
| Qatar | 0.1(-0.15 to 0.42) | 0.18(-0.02 to 0.4) | -0.98(-0.99 to -0.95) | -0.98(-0.99 to -0.95) |
| Republic of Korea | -0.04(-0.35 to 0.6) | 0.03(-0.22 to 0.62) | -0.98(-0.99 to -0.95) | -0.98(-0.99 to -0.95) |
| Republic of Moldova | -0.12(-0.49 to 0.73) | 0.36(-0.2 to 1.6) | -0.9(-0.95 to -0.83) | -0.85(-0.93 to -0.74) |
| Romania | 0.05(-0.33 to 1) | 0.43(-0.06 to 1.66) | -0.76(-0.9 to -0.57) | -0.68(-0.86 to -0.43) |
| Russian Federation | 0.29(-0.22 to 1.28) | 0.05(-0.35 to 0.8) | -0.65(-0.86 to -0.31) | -0.71(-0.88 to -0.41) |
| Rwanda | 0.57(0 to 2.11) | 0.6(0.03 to 2.1) | -0.41(-0.6 to -0.17) | -0.4(-0.59 to -0.19) |
| Saint Kitts and Nevis | 0.07(-0.59 to 2.53) | 0.25(-0.51 to 3.12) | -0.89(-0.96 to -0.72) | -0.87(-0.96 to -0.67) |
| Saint Lucia | 0.45(-0.18 to 2.66) | 0.76(-0.01 to 3.4) | -0.94(-0.97 to -0.88) | -0.92(-0.96 to -0.85) |
| Saint Vincent and the Grenadines | 0.84(0.04 to 4.31) | 1.1(0.21 to 4.93) | -0.89(-0.95 to -0.81) | -0.88(-0.94 to -0.78) |
| Samoa | 0.64(-0.17 to 2.77) | 0.78(-0.09 to 3.04) | 0.01(-0.26 to 0.38) | 0.09(-0.17 to 0.42) |
| San Marino | -0.34(-0.72 to 1.11) | 0.15(-0.47 to 2.5) | -0.82(-0.93 to -0.54) | -0.69(-0.88 to -0.22) |
| Sao Tome and Principe | 3.13(1.42 to 7.5) | 3.46(1.74 to 7.84) | -0.22(-0.5 to 0.1) | -0.15(-0.44 to 0.13) |
| Saudi Arabia | 0.52(-0.07 to 1.86) | 1.3(0.51 to 3.15) | -1(-1 to -0.99) | -1(-1 to -0.98) |
| Senegal | 1.48(0.67 to 3.61) | 1.55(0.77 to 3.69) | 0.02(-0.28 to 0.41) | 0.04(-0.25 to 0.34) |
| Serbia | 0.5(0.01 to 1.55) | 0.66(0.18 to 1.77) | -0.64(-0.83 to -0.33) | -0.59(-0.81 to -0.28) |
| Seychelles | 1.1(0.69 to 1.74) | 1.47(1.13 to 2.1) | -0.9(-0.96 to -0.81) | -0.89(-0.95 to -0.77) |
| Sierra Leone | 1.38(0.61 to 3.32) | 1.58(0.75 to 3.67) | 0.14(-0.18 to 0.59) | 0.22(-0.11 to 0.67) |
| Singapore | -0.87(-0.91 to -0.7) | -0.4(-0.61 to 0.3) | -1(-1 to -0.99) | -0.98(-0.99 to -0.97) |
| Slovakia | -0.44(-0.62 to -0.11) | -0.13(-0.36 to 0.36) | -0.85(-0.95 to -0.65) | -0.77(-0.92 to -0.43) |
| Slovenia | -0.4(-0.62 to 0.03) | -0.07(-0.33 to 0.46) | -0.79(-0.92 to -0.56) | -0.68(-0.87 to -0.34) |
| Solomon Islands | 3.09(0.92 to 10.63) | 3.12(0.95 to 10.47) | 0.51(-0.01 to 1.2) | 0.51(0.03 to 1.11) |
| Somalia | 0.32(0.01 to 0.82) | 0.41(0.1 to 0.88) | -0.05(-0.27 to 0.27) | 0(-0.21 to 0.28) |
| South Africa | 1.19(0.88 to 1.63) | 1.11(0.82 to 1.49) | -0.43(-0.62 to -0.19) | -0.51(-0.67 to -0.31) |
| South Sudan | 0.72(0.16 to 1.9) | 0.82(0.29 to 2.05) | -0.19(-0.42 to 0.12) | -0.13(-0.35 to 0.15) |
| Spain | -0.65(-0.8 to -0.21) | -0.32(-0.63 to 0.58) | -0.94(-0.98 to -0.87) | -0.89(-0.96 to -0.75) |
| Sri Lanka | 3.47(1.37 to 9.07) | 3.23(1.31 to 8.38) | 0.26(-0.27 to 0.93) | 0.17(-0.3 to 0.71) |
| Sudan | 5.49(2.3 to 17.4) | 6.94(3.22 to 20.12) | -0.47(-0.73 to -0.09) | -0.35(-0.66 to 0.05) |
| Suriname | 0.8(-0.05 to 3.64) | 1.09(0.12 to 4.28) | -0.68(-0.84 to -0.41) | -0.63(-0.81 to -0.32) |
| Sweden | -0.66(-0.91 to 0.24) | -0.55(-0.89 to 0.64) | -0.88(-0.97 to -0.69) | -0.85(-0.97 to -0.59) |
| Switzerland | -0.72(-0.83 to -0.48) | -0.46(-0.68 to -0.01) | -0.85(-0.93 to -0.66) | -0.7(-0.87 to -0.32) |
| Syrian Arab Republic | 0(-0.28 to 0.48) | 0.35(0.1 to 0.72) | -0.99(-1 to -0.98) | -0.99(-1 to -0.97) |
| Taiwan (Province of China) | 0.2(-0.23 to 1.18) | 0.36(-0.08 to 1.48) | -0.91(-0.97 to -0.8) | -0.9(-0.96 to -0.78) |
| Tajikistan | 5.28(2.54 to 13.09) | 4.44(2.18 to 10.68) | 0.21(-0.3 to 0.85) | 0.05(-0.35 to 0.54) |
| Thailand | 0.31(-0.25 to 1.73) | 0.75(0.05 to 2.57) | -0.79(-0.91 to -0.62) | -0.72(-0.87 to -0.51) |
| Timor-Leste | 2.61(0.65 to 9.78) | 3.37(1.04 to 11.96) | -0.19(-0.51 to 0.16) | -0.01(-0.39 to 0.34) |
| Togo | 1.44(0.61 to 3.42) | 1.56(0.73 to 3.58) | 0(-0.31 to 0.38) | 0.04(-0.27 to 0.37) |
| Tokelau | 0.14(-0.91 to 42.1) | 0.2(-0.9 to 44.63) | -0.91(-1 to 0.67) | -0.91(-1 to 0.72) |
| Tonga | 1.52(0.25 to 5.16) | 1.65(0.3 to 5.38) | -0.37(-0.62 to -0.07) | -0.34(-0.59 to -0.04) |
| Trinidad and Tobago | -0.13(-0.49 to 1.35) | -0.08(-0.43 to 1.41) | -0.92(-0.97 to -0.8) | -0.92(-0.97 to -0.78) |
| Tunisia | 0.78(0.24 to 1.63) | 1.18(0.78 to 1.88) | -0.98(-0.99 to -0.96) | -0.98(-0.99 to -0.95) |
| Turkey | -0.26(-0.44 to 0.08) | -0.01(-0.22 to 0.35) | -0.99(-1 to -0.97) | -0.98(-0.99 to -0.96) |
| Turkmenistan | 0.83(0.23 to 2.29) | 0.93(0.38 to 2.42) | -0.88(-0.95 to -0.69) | -0.87(-0.95 to -0.68) |
| Tuvalu | 2.4(0.19 to 9.06) | 2.52(0.23 to 9.35) | -0.74(-0.92 to -0.48) | -0.73(-0.91 to -0.47) |
| Uganda | 2.11(0.83 to 6.82) | 2.38(1.02 to 7.4) | -0.22(-0.51 to 0.14) | -0.15(-0.46 to 0.17) |
| Ukraine | -0.29(-0.54 to 0.18) | -0.04(-0.37 to 0.6) | -0.73(-0.89 to -0.46) | -0.62(-0.83 to -0.24) |
| United Arab Emirates | -0.26(-0.52 to 0.04) | -0.01(-0.24 to 0.25) | -0.99(-1 to -0.99) | -0.99(-1 to -0.98) |
| United Kingdom | -0.69(-0.81 to -0.41) | -0.05(-0.44 to 0.85) | -0.87(-0.95 to -0.72) | -0.64(-0.86 to -0.22) |
| United Republic of Tanzania | 1.63(0.72 to 4.14) | 1.93(0.92 to 4.59) | -0.15(-0.41 to 0.14) | -0.05(-0.34 to 0.23) |
| United States of America | -0.53(-0.76 to 0.01) | -0.37(-0.68 to 0.34) | -0.64(-0.86 to -0.25) | -0.51(-0.82 to -0.01) |
| United States Virgin Islands | 0.08(-0.61 to 6.27) | 0.3(-0.53 to 7.56) | -0.83(-0.94 to -0.5) | -0.79(-0.93 to -0.39) |
| Uruguay | -0.04(-0.56 to 2.08) | 0.45(-0.32 to 3.56) | -0.85(-0.93 to -0.71) | -0.77(-0.9 to -0.57) |
| Uzbekistan | 5.75(3.2 to 13.22) | 4.26(2.38 to 10.21) | -0.05(-0.57 to 0.86) | -0.26(-0.67 to 0.44) |
| Vanuatu | 2.13(0.56 to 7.05) | 2.2(0.62 to 6.84) | 0.56(0.04 to 1.23) | 0.58(0.14 to 1.08) |
| Venezuela (Bolivarian Republic of) | 0.35(-0.14 to 1.57) | 0.43(-0.03 to 1.72) | -0.88(-0.95 to -0.72) | -0.87(-0.94 to -0.7) |
| Viet Nam | 2.52(0.69 to 8.22) | 2.92(0.93 to 9.21) | -0.51(-0.72 to -0.23) | -0.45(-0.68 to -0.18) |
| Yemen | 4.37(1.61 to 14.61) | 5.31(2.3 to 16.96) | -0.47(-0.71 to -0.1) | -0.37(-0.65 to 0) |
| Zambia | 1.75(0.65 to 4.54) | 1.93(0.8 to 4.88) | -0.27(-0.51 to 0.01) | -0.22(-0.48 to 0.03) |
| Zimbabwe | 1.13(0.55 to 2) | 1.2(0.68 to 1.99) | 0.38(0.03 to 0.77) | 0.44(0.15 to 0.74) |

Abbreviations: APMP ambient particulate matter pollution; HAP household air pollution; SDI socio-demographic index; GBD global burden of disease; ASDR age-standardized death rate; DALY disability-adjusted life year; UI uncertainty interval.
